# Allogeneic hematopoietic cell transplantation in mature T- or NK-lymphomas: a phase II clinical trial

**DOI:** 10.1038/s41467-026-71461-5

**Published:** 2026-04-23

**Authors:** Kamil A. Rechache, Natalia S. Nunes, Jennifer Sponaugle, Elisabetta Xue, Amy Chai, Rebecca Combs, Jessenia Campos, Xingmin Feng, Francis A. Flomerfelt, Kalpana Upadhyaya, William Telford, Brian Dawson, Thomas E. Hughes, Syed Muhammad Salman Shah, Alicia Peluso, Christi McKeown, Anita Stokes, Scott Napier, Ruby Sabina, Jennifer Wilder, Kristen Cole, Judy Baruffaldi, Jennifer Cuellar-Rodriguez, Juan Gea-Banacloche, Mary M. Czech, Mustafa A. Hyder, Christopher G. Kanakry, Dimana Dimitrova, Jennifer A. Kanakry

**Affiliations:** 1 Center for Immuno-Oncology, Center for Cancer Research, National Cancer Institute, National Institutes of Health, Bethesda, MD USA; 2 Hematology Branch, National Heart, Lung, and Blood Institute, National Institutes of Health, Bethesda, MD USA; 3 Laboratory of Pathology, Center for Cancer Research, National Cancer Institute, National Institutes of Health, Bethesda, MD USA; 4 Department of Laboratory Medicine, Clinical Center, National Institutes of Health, Bethesda, MD USA; 5 Clinical Pharmacology Program, Center for Cancer Research, National Cancer Institute, National Institutes of Health, Bethesda, MD USA; 6 Office of the Clinical Director, Center for Cancer Research, National Cancer Institute, National Institutes of Health, Bethesda, MD USA; 7 Frederick National Laboratory for Cancer Research, National Cancer Institute, National Institutes of Health, Bethesda, MD USA; 8 Immune Deficiency and Cellular Therapy Program, Center for Cancer Research, National Cancer Institute, National Institutes of Health, Bethesda, MD USA; 9 Office of the Chief Medical Officer, Clinical Center, National Institutes of Health, Bethesda, MD USA; 10 National Institute of Allergy and Infectious Diseases, NIH, Bethesda, MD USA

**Keywords:** Stem-cell research, T-cell lymphoma, Cancer therapy, Phase II trials

## Abstract

Allogeneic hematopoietic cell transplantation (HCT) is a potential cure for patients with peripheral T-/NK-cell lymphomas (PTCL), but its application is understudied. This prospective trial evaluates a unique reduced-intensity (RIC) transplantation platform in 31 patients with PTCL (NCT03922724). One-year progression-free survival, the primary endpoint, is 53% (95% CI 29-72%) on the RIC arm and 60% (95% CI 25-83%) on the modified-RIC arm. The 3-year overall survival is 61% (95% CI 42-76%), with relapse estimated at 18% (95% CI 6-34%) at 3 years. Transplant-related mortality was 24% (95% CI 10-41%) at 1-year, low at 11% (95% CI 2-29%) for patients ≤60 years but 56% (95% CI 17-82%) for patients >60 years, *p* = 0.01. There was no grade III-IV acute graft-versus-host disease, while chronic graft-versus-host disease was estimated at 23% (95 CI 10-38%) at 2 years. This study demonstrates a benefit regardless of pre-transplantation disease status, challenging the requirement of remission for HCT.

## Abstract (web summary)

Allogeneic haematopoietic cell transplantation is a potential therapeutic strategy for patients with mature T-/NK-cell lymphoma. Here the authors present the results of a phase II clinical trial of allogeneic haematopoietic cell transplantation for mature T or NK-cell lymphoma and suggest induction of graft versus tumor immunity is linked to remission even in the context of refractory disease.

## Introduction

Peripheral (mature) T-and natural killer (NK)-cell lymphomas (PTCL) are a small subset of non-Hodgkin lymphomas that tend to be aggressive and poorly responsive to chemotherapy^1–10^. In addition to suboptimal response rates to first-line therapy, the majority of patients with PTCL are unable to achieve a complete response (CR) after relapse, despite the emergence of scientifically-promising drug combinations^4,11,12^. Intensified chemotherapy-based approaches have not improved cure rates and can be met with significant toxicities; consolidative autologous stem cell transplant requires chemosensitive disease^13,14^.

Given this profile, cellular immunotherapy is a main avenue to improve outcomes for patients with PTCL. Allogeneic hematopoietic cell transplantation (HCT) is a potentially curative cellular immunotherapy option for relapsed/refractory PTCL or as front-line consolidation for certain high-risk subtypes^12,15,16^. Even so, those with PTCL who reach HCT face high rates of transplant-related mortality (TRM) and post-HCT relapse. Furthermore, these results include only those who had access to and were deemed eligible for HCT, excluding those with prohibitive comorbidities or with aggressive disease outpacing timely referral or rendering them ineligible due to chemorefractoriness^14,17–22^. Meanwhile, the application of chimeric antigen receptor T-cell (CART) therapy to PTCL remains challenging due to the lack of T-cell neoplasm-specific target antigens, T-cell fratricide, manufacturing impurities, the dangers of longstanding T-cell aplasia, and possibly CART-related malignancies^23–25^.

The American Society for Transplantation and Cellular Therapy (ASTCT) has identified a need for prospective, PTCL-specific HCT trials^16^, yet at present, the study presented herein is only one of two current prospective trials^26,27^.

We present the results of a prospective trial of HCT for patients with PTCL using a unique reduced-intensity conditioning (RIC) approach incorporating distally-timed horse antithymocyte globulin (hATG), T-cell replete peripheral blood stem cell (PBSC) grafts, and post-transplantation cyclophosphamide (PTCy)-based GVHD prophylaxis with abbreviated adjunct immunosuppression ending at day +60. Unlike prior studies, disease control was not required for eligibility, human leukocyte antigen (HLA)-mismatched donors were heavily utilized, and there was a low threshold to further abbreviate GVHD prophylaxis to minimize toxicities and/or to promote immune reconstitution (IR) and graft-vs.-tumor (GVT) activity.

## Results

### Patient, disease, and graft characteristics

**Fig. 1:**
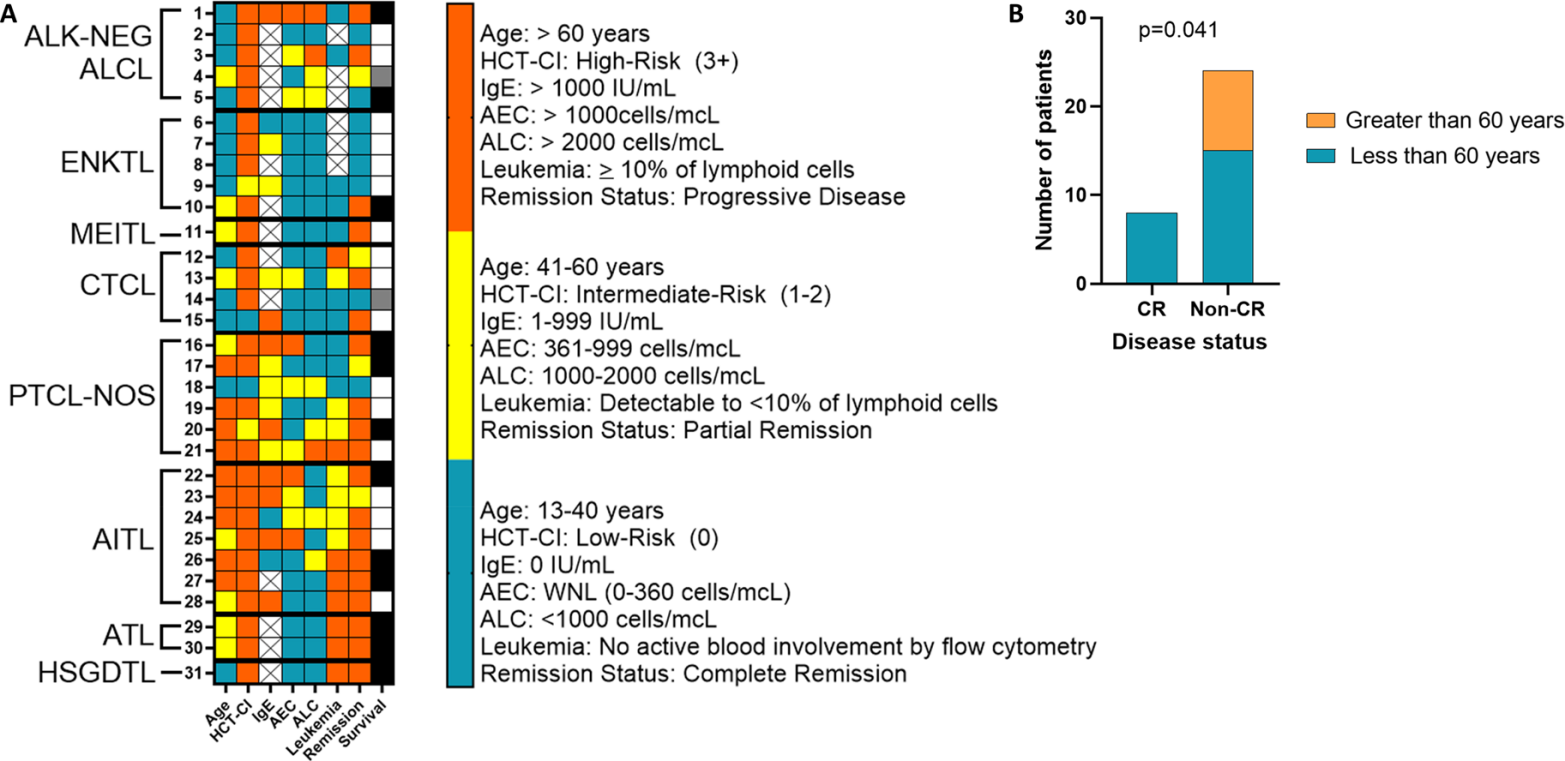
Pre-HCT patient and disease characteristics. **A** Pre-HCT patient and disease characteristics, by patient, grouped by histologic subtype and stratified into favorable (blue), intermediate (yellow), and adverse (orange) characteristics and survival outcomes (death, black; alive with progression/relapse, gray; alive without progression/relapse, white). **B** Pre-HCT disease status by age. Chi-square test (two-sided) shows statistical significance. Missing data are marked, X.

**Fig. 2:**
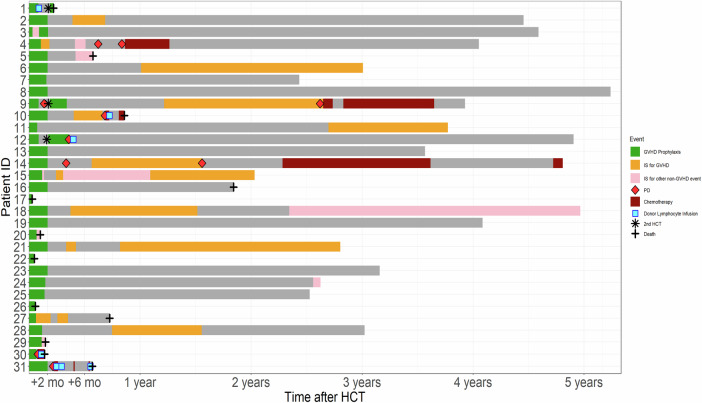
Swimmer’s plot of individual outcomes. Post-HCT survival duration shown in gray bars, with GVHD prophylaxis (teal) and treatment with immunosuppression (IS, yellow or pink, depending on indication) noted. Patient IDs match the patient IDs in Fig. 1, so that the patients are organized by disease histology, not by chronologic enrollment. Key events and interventions post-HCT are shown, including relapse/progression (red diamond), donor lymphocyte infusion (blue square), second HCT (asterisk, all for graft failure), and death (cross). Periods of chemotherapy for relapse/progression are shown (orange), notably not required immediately upon relapse/progression for some.

Thirty-one patients were enrolled on this study, of which 21 were transplanted on the RIC arm, followed by 10 patients on the modified RIC (mRIC) arm, Table 1. The majority of grafts (68%) were from HLA-mismatched, especially HLA-haploidentical (*n* = 19, 61%) donors. Unrelated donor grafts (*n* = 9) were also critical for enabling HCT in this study, overcoming barriers of adoption with no known biologic family, family estrangement, or donor-specific anti-HLA antibodies. Several poor-prognosis, very rare histologic subtypes were included, although 18 patients had the more common subtypes of angioimmunoblastic T cell lymphoma (AITL, *n* = 7), PTCL-not otherwise specified (NOS, *n* = 6), and ALK-negative anaplastic large cell lymphoma (ALCL, *n* = 5). Pre-HCT disease histology, remission status, and associated disease features are mapped individually by patient in Fig. 1A. The majority of patients (*n* = 19, 61%) had refractory, active disease (less than partial remission [PR]) immediately pre-HCT. No patients older than age 60 years entered HCT in CR, *p* = 0.04, Fig. 1B. It should be noted that even patients deemed to be in remission at pre-HCT evaluations were not observed off therapy to determine if pre-HCT remission was durable, but rather were tightly bridged from salvage therapy to HCT. Figure 2 outlines comprehensive time courses of follow-up for each patient, with delineated periods of GVHD prophylaxis, treatment with systemic immunosuppression, and post-HCT chemotherapy, as well as events of second HCT, donor lymphocyte infusion (DLI), disease relapse/progression, and death. Supplementary Table 1 outlines the details of individual patient and donor demographics, graft dose, and outcomes.

**Table 1:** Patient and graft characteristics

Characteristic	All patients (*n* = 31)	RIC (*n* = 21)	mRIC (*n* = 10)
HCT timeframe, month/year	5/2019–8/2022	5/2019–7/2021^a^	7/2021–8/2022
Male sex, *n* (%)	15 (48%)	11 (52%)	4 (40%)
Age at HCT, median (range), yr	46 (13–71)	44 (22–71)	60 (13–67)
Race/ethnicity, *n* (%)			
Black/African descent	13 (42%)	8 (38%)	5 (50%)
Caucasian, non-Hispanic	9 (29%)	6 (29%)	3 (30%)
Caucasian, Hispanic	8 (26%)	7 (33%)	1 (10%)
Asian	1 (3%)	0	1 (10%)
Histopathologic subtype, *n* (%)			
AITL	7 (23%)	2 (10%)	5 (50%)
PTCL-NOS	6 (19%)	4 (19%)	2 (20%)
ALK-negative ALCL	5 (16%)	4 (19%)	1 (10%)
ENKTCL	5 (16%)	4 (19%)	1 (10%)
CTCL	4 (13%)	3 (14%)	1 (10%)
ATL	2 (6%)	2 (10%)	0
HSGDTCL	1 (3%)	1 (5%)	0
MEITL	1 (3%)	1 (5%)	0
Disease status at HCT, *n* (%)			
Progressive disease	19 (61%)	10 (48%)	9 (90%)
CR1 after primary refractory	4 (13%)	3 (14%)	1 (10%)
CR2	4 (13%)	4 (19%)	0 (0%)
PR1 after primary refractory	1 (3%)	1 (5%)	0 (0%)
PR2 or beyond	3 (10%)	3 (14%)	0 (0%)
Prior lines of therapy, median (range)	3 (1–14)	3 (1–6)	4–5 (1–14)
Prior autologous stem cell transplant, *n* (%)	4 (13%)	2 (10%)	2 (20%)
CNS involvement, *n* (%)	5 (16%)	3 (14%)	2 (20%)
HCT-CI, median (range)	4 (0–9)	4 (0–7)	4 (0–9)
Donor age, median (range), yr	31 (15–53)	31 (20–53)	30 (15–40)
Female donor for male recipient, *n* (%)	4 (13%)	3 (14%)	1 (10%)
Donor match, *n* (%)			
HLA-haploidentical	19 (61%)	13 (62%)	6 (60%)
HLA-matched unrelated	7 (23%)	4 (19%)	3 (30%)
HLA-matched sibling	3 (10%)	3 (14%)	0
HLA-mismatched unrelated	2 (6%)	1 (5%)	1 (10%)
ABO, *n* (%)			
Matched	16 (52%)	13 (62%)	3 (30%)
Major mismatch	7 (23%)	5 (24%)	2 (20%)
Minor mismatch	6 (19%)	2 (10%)	4 (40%)
Major and minor mismatch	2 (6%)	1 (5%)	1 (10%)
Graft dose, median (range), cells/kg			
TNC, × 10^8^	9.6 (4.5–18.4)	9.3 (4.5–18.4)	12.0 (6.6–16.2)
CD3^+^, × 10^7^	29.0 (11.8–68.6)	27.9 (11.8–56.4)	36.3 (19.4–68.6)
CD34^+^, × 10^6^	10.0 (7.1–13.8)	10.0 (7.1–13.8)^b^	10.0 (7.8–12.8)
CMV serostatus D/R, *n* (%)			
+/+	16 (52%)	9 (43%)	7 (70%)
−/+	5 (16%)	3 (14%)	2 (20%)
−/−	5 (16%)	4 (19%)	1 (10%)
+/−	5 (16%)	5 (24%)	0

^a^One additional patient was transplanted per the RIC arm on 4/15/2022 despite enrolling on the mRIC arm, upon discovery on day −7 that filgrastim on days −12 and −8 had not been given.

^b^One patient was found to have very low viability of the cryopreserved graft upon thaw on HCT day, with the decision to give all the cells available, totaling 24 × 10^6^ CD34^+^/kg total cells, with a viability ranging from 21–37%, among the tested bags. This dose is not included in the table values above, given the outlier nature and specifics. Unfortunately, this patient experienced secondary graft failure.

### Survival and relapse endpoints

**Fig. 3:**
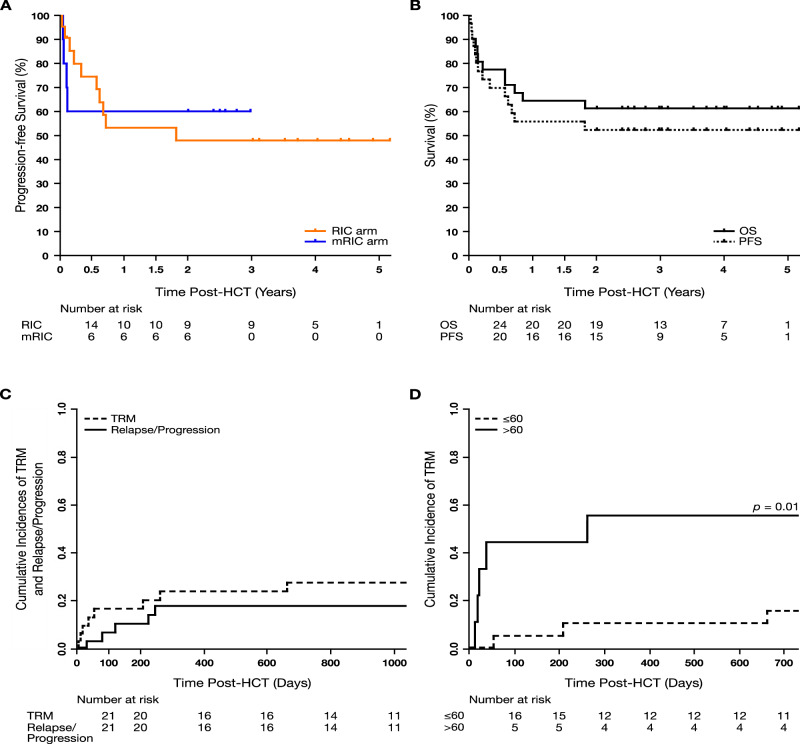
Survival and relapse endpoints. **A** Kaplan-Meier survival curve of PFS by arm. **B** Kaplan-Meier survival curve of PFS and OS, for the whole cohort. **C** Cumulative incidence of relapse and TRM, for the cohort. **D** Cumulative incidence of TRM, by age in years, compared by Gray’s test.

With median follow-up of 3.5 years for surviving patients, the primary endpoint of 1-year progression-free survival (PFS) was 53% (95% confidence interval (CI) 29–72%) for the RIC arm and 60% (95% CI 25–83%) for the mRIC arm (Fig. 3A). For the entire cohort, the estimated 3-year PFS was 52% (95% CI 33–68%), with 3-year overall survival (OS) of 61% (95% CI 42–76%), Fig. 3B, with additional exploratory subgroup survival analyses in Table 2, including by disease type, disease status at HCT, HLA-match, age, and HCT-comorbidity index (HCT-CI) score.

**Table 2:** Exploratory subgroup analyses of survival outcomes

	1-year PFS	1-year OS	3-year PFS	3-year OS
All patients (*n* = 31)	56%	65%	52%	61%
Excluding ATL (*n* = 29)	60%	69%	56%	66%
RIC arm (*n* = 21)	53%	67%	48%	67%
RIC arm, excluding ATL (*n* = 19)	60%	74%	56%	68%
mRIC (*n* = 10)	60%	60%	60%	60%
By degree of HLA match				
HLA-mismatched (*n* = 21)	43%	57%	38%	52%
HLA-haplo, recipient age ≤ 60 years (*n* = 14)	59%	79%	51%	71%
HLA-matched (*n* = 10)	80%	80%	80%	80%
By recipient age				
Age ≤ 60 years (*n* = 22)	60%	73%	55%	68%
Age > 60 years (*n* = 9)	44%	44%	44%	44%
By disease type				
PTCL-NOS, AITL, ALK-neg ALCL (*n* = 18)	56%	61%	50%	56%
Other (*n* = 13)	55%	69%	55%	69%
By HCT-CI score				
HCT-CI 4+ (*n* = 18)	56%	61%	50%	56%
HCT-CI ≤ 3 (*n* = 13)	56%	69%	56%	69%
By pre-HCT disease status				
Active disease (*n* = 19)	53%	53%	47%	47%
PR/CR (*n* = 12)	61%	83%	61%	83%

*PFS* progression-free survival, *OS* overall survival, *ATL* adult T-cell leukemia/lymphoma, *RIC* reduced-intensity conditioning, *mRIC* modified RIC, *HLA* human leukocyte antigen, *PTCL-NOS* peripheral T cell lymphoma not otherwise specified, *AITL* angioimmunoblastic T cell lymphoma, *ALK-neg* ALCL, ALK negative anaplastic large cell lymphoma, *HCT-CI* hematopoietic cell transplantation comorbidity index, *PR* partial remission, *CR* complete remission, *haplo* haploidentical.

The 3-year cumulative incidence (CuI) of relapse was 18% (95% CI 6–34%) for all patients (Fig. 3C) and 16.5% (95% CI 4–37%) for patients with active disease (*n* = 19, those with less than PR) at the time of HCT. The CuI of TRM at day 100 and 1-year was 17% (95% CI 6–32%) and 24% (95% CI 10–41%), respectively, for all patients, Fig. 3C. Notably, the Cul of TRM at 1-year was 11% (95% CI 2–29%) for patients ≤60 years but significantly higher at 56% (95% CI 17–82%) for patients >60 years, *p* = 0.01 (Fig. 3d), the latter exclusively in recipients of HLA-mismatched grafts, who also had greater comorbidities and were more heavily pretreated. Causes of death are outlined in Table 3, with early cardiopulmonary mortalities in a notable subset, particularly older patients.

**Table 3:** Causes of death, in chronologic order

Arm	Donor source	Disease	Patient age	Cause of death	Days post-HCT	Timeframe	Main organ	Autopsy diagnosis
RIC	MSD^a^	ATL	51	Respiratory failure, due to post-engraftment, non-infectious lung process	Day +53	1/2020	Lung	Lipoid and organizing pneumonia (also seen on pre-mortem VATS on day +36)
RIC	Haplo	AITL	65	Sepsis (*Staphylococcus aureus)*, on outpatient parenteral nutrition due to malnutrition after gut acute GVHD and infectious enterocolitis	Day +260	2/2020	Line-associated infection	Not performed
RIC	Haplo^b^	ATL	47	Disease progression	Day +50	3/2020	Disease	Diffuse, multi-organ involvement by lymphoma
RIC	Haplo	HSGDTCL	35	Disease progression	Day +205	8/2020	Disease	Not performed
RIC	MSD	ENKTCL	45	Disease progression, occurring in the setting of treatment for acute GVHD	Day +309	1/2021	Disease	Not performed
RIC	7/8 URD	ALK-neg ALCL	38	SARS-CoV2 vaccine-related lymphocytic interstitial pneumonitis (reported to VAERS)	Day +207	5/2021	Lung	Fibrotic stage diffuse alveolar damage
mRIC	Haplo	AITL	61	Brain death, after witnessed PEA arrest with inability to secure advanced airway	Day +17	9/2021	Heart	Not performed
RIC	Haplo	PTCL-NOS	56	Cardiac arrest while performing iADLs, with medical co-morbidities of DM2, HTN, HLD	Day +663	10/2021	Heart	Not performed
mRIC	Haplo	ALK-neg ALCL	13	Cardiac arrest, during engraftment, off study after 2nd HCT (had graft failure on study)	Day +79 (after 2nd HCT)	12/2021	Heart	Not performed
mRIC	Haplo	PTCL-NOS	71	Sepsis	Day +10	4/2022	Infection	*Enterobacter cloacae* complex bacteremia
mRIC	Haplo	PTCL-NOS	67	Engraftment syndrome, polymicrobial sepsis resuscitation contributing	Day +36	9/2022	Lung	Pulmonary edema with ARDS
mRIC	Haplo	AITL	65	Engraftment syndrome, polymicrobial sepsis resuscitation contributing	Day +20	9/2022	Lung	Not performed

*RIC* reduced intensity conditioning, *mRIC* modified reduced intensity conditioning, *HCT* hematopoietic cell transplantation, *VATS* video-assisted thoracic surgery, *SARS-CoV2* severe acute respiratory syndrome related coronavirus 2, *GVHD* graft versus host disease, *VAERS* vaccine adverse event reporting system, *PEA* pulseless electrical activity, *iADLs* instrumental activities of daily living, *DM2* type II diabetes mellitus, *HTN* hypertension, *HLD* hyperlipidemia, *ARDS* acute respiratory distress syndrome.

^a^Donor HTLV-1 seronegative. Donor diagnosed with JAK2V617F myeloproliferative neoplasm in the year post-donation.

^b^Donor HTLV-1 seropositive. Donor diagnosed with lymphomatous subtype, HTLV-1-associated adult T cell leukemia in the year post-donation.

### Engraftment and chimerism

The median time to neutrophil engraftment was 17 days (range 12–39). The 60-day CuI of GF was 10% (95% CI 2–23%), with instances of GF being associated with rebound of donor-specific anti-HLA antibodies (*n* = 1)^28^, and low-viability cryopreserved graft (*n* = 1, Table 1 footnote); both were re-transplanted off-study, with ongoing survival. A third GF was in the setting of extreme immune dysregulation from refractory hypereosinophilic syndrome and could not be stabilized with DLI; this patient died shortly after the second, off-study HCT. Poor graft function occurred in one patient, now with adequate graft function after an unconditioned, CD34^+^-selected stem cell boost on day +128.

**Fig. 4:**
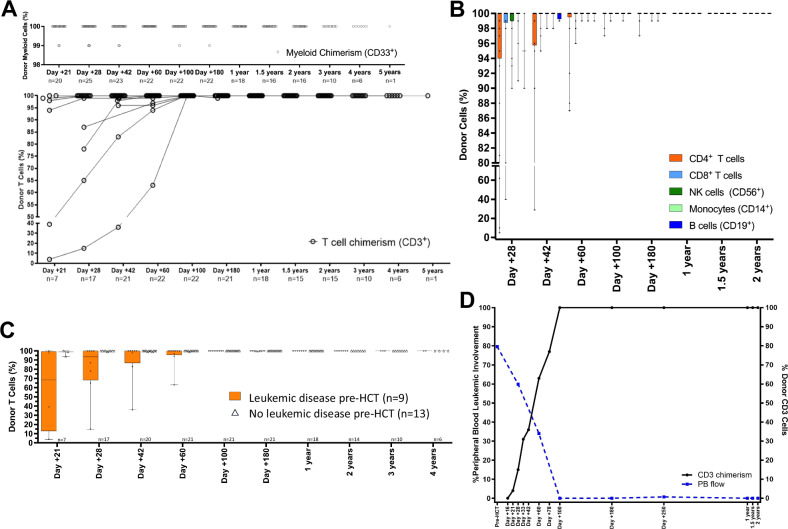
Chimerism, expressed as percentage of donor cells in peripheral blood for evaluable patients, *n* = 22. Chimerism analysis required sufficient numbers of cells to assay, affecting sample size at each timepoint, with sample size also subject to changes in survival and follow-up duration at each timepoint. **A** Myeloid and CD3+ T cell chimerism over time in engrafted patients, **B** lineage-specific cell subset chimerism over time, **C** CD3+ T cell chimerism over time, by pre-HCT peripheral blood (leukemic) disease involvement. Box and whisker plots indicate minimum, first quartile, median, third quartile, and maximum. Each symbol represents an individual patient data point. **D** Single patient example of the inverse relationship between achieving full donor T cell chimerism and clearance of leukemic disease, quantified in peripheral blood by flow cytometry.

Serial chimerism analysis demonstrated early and robust achievement of donor myeloid chimerism, with all engrafting patients achieving and maintaining 99–100% donor myeloid cells by day +21 (Fig. 4A). In the T-cell compartment, CD3^+^ chimerism was overall robust, but slower to reach 100% donor compared to myeloid chimerism (Fig. 4A), particularly in those with active, T-cell leukemic disease in the peripheral blood (Fig. 4C). The kinetics of achieving 100% donor T-cell chimerism was dramatically inversely related to post-HCT leukemic disease status by flow cytometry in the peripheral blood in one patient (Fig. 4D). Lineage-specific chimerism showed robust engraftment of T cells (CD4^+^, CD8^+^), NK cells (CD56^+^), B cells (CD19^+^), and monocytes (CD14^+^), Fig. 4B, but highlights the slower rise of CD4+ T-cell chimerism, again mirroring largely CD4^+^ leukemic disease early post-HCT.

### GVHD

Notably, no patients developed grade III-IV acute GVHD (aGVHD). The 1-year Cul of grades I–II aGVHD was 19% (95% CI 8–35%), with 1-year CuI of 20% (95% CI 3–48%) with HLA-matched grafts and 19% (95% CI 6–39%) with HLA-mismatched grafts. Moreover, the 1-year Cul of grade II aGVHD was 16% (95% CI 6–31%) for all patients. Chronic GVHD (cGVHD) had a 2-year CuI of 23% (95 CI 10–38%), all with maximal global severity of severe, with onset post-HCT at a median 184 days (range 99–945), with 2-year Cul of cGVHD of 30% (95% CI 7–58%) for recipients of HLA-matched grafts and 19% (95% CI 6–37%) for HLA-mismatched grafts.

### Graft versus tumor effect

There was a low rate of relapse/progression despite the high number of patients with active disease at the time of HCT (Fig. 1), highlighting the role of GVT in HCT for PTCL. To cultivate GVT, immunosuppression was not escalated beyond GVHD prophylaxis whenever possible. The duration of GVHD prophylaxis in the study was abbreviated, with mycophenolate mofetil ending at day +25 and sirolimus ending at day +60 without taper. Beyond that abbreviated schedule, sirolimus was stopped earlier than the planned day +60 in 11 (35%) patients. Figure 2 demonstrates the timing between immunosuppression and disease re-emergence, notably for P4, P9, P12, and P14. DLI was given to four patients in the setting of disease progression, with one (Fig. 2, Pt 12) responding durably to DLI after second HCT and another (Fig. 2, patient 31) with remarkable, clinically evident regression/resolution of cutaneous lesions, but unfortunately mixed and ultimately only transient response before fatal, aggressive disease progression^29^.

Several patients had significant leukemic involvement (*n* = 12), with median 14% (range 0.3–83%) of lymphoid cells in peripheral blood being malignant immediately pre-HCT, which could be readily followed by flow cytometry serially post-HCT (Supplemental Fig. 1A). Given the intermittent detection of subclinical disease by flow cytometry, flow cytometric disease monitoring did inform the use of post-HCT immunosuppression and the risk of hindering GVT. Many patients had presumed pleural/lung involvement by lymphoma pre-HCT, with low adjusted diffusing capacity (DLco), waxing/waning nodular findings, and/or pleural effusions on pre-HCT imaging, although six had pathology-confirmed lung/pleural involvement. Post-HCT serositis was a presumed GVT phenomenon seen mainly in patients with AITL (*n* = 4)^8^, although also in one patient with PTCL-NOS. Post-HCT serositis occurred most commonly as chronic, bilateral pleural effusions (*n* = 5) with intermittent detection of lymphoma by flow cytometry of pleural fluid (Supplemental Fig. 1B), but also as ascites (*n* = 1), ear effusions (*n* = 2), and pericardial effusion (*n* = 1). This posed significant morbidity with the need for thoracentesis (*n* = 5), as well as pleural catheters and/or chest tubes for large volume, routine drainage of effusions for 2-, 5.5-, and 12-months for 3 patients. Other interventions included emergency pericardial fluid drainage for tamponade (*n* = 1), large volume paracentesis (*n* = 1 patient, 4 procedures), and myringotomy with pressure-equalization tube placement (*n* = 2). However, the effusions resolved with this supportive care and monitoring, with the preference to observe, support, and not treat with steroids or other immunosuppression, respecting the process of what was deemed to be likely an ongoing (and, in each case, ultimately successful) GVT phenomenon.

Most patients were evaluated pre-HCT for central nervous system (CNS) involvement with brain MRI and cerebrospinal fluid (CSF) flow cytometric, cytopathologic, and molecular analysis, with five being found at HCT screening to be positive in CSF (*n* = 4 were asymptomatic). All five had chemorefractory systemic disease that could not tolerate HCT delay for long; HCT was delayed until CSF cleared in two patients, resulting in 54- and 115-day delays, but could not be delayed further in the remaining three, who went to HCT with flow cytometric-positive CSF after delays of 28-, 44-, and 70-days of CNS-directed therapies. Of the three who proceeded with CSF positivity, one cleared on day −6, while the other two remained positive during the twice-weekly intrathecal chemotherapy given during conditioning. Of these two, follow-up was limited by early TRM for one, while the other remarkably has had no post-HCT CSF disease, despite no post-HCT CNS-directed therapy, as of 3-years post-HCT, providing remarkable evidence of GVT in the CNS. Post-HCT CNS-directed therapy was specifically not given prophylactically, so as to not impair any possible GVT effect, nor did the need arise to treat for CNS symptoms.

### Complications and toxicities

Many patients were high-risk with significant co-morbidities, where the median HCT-CI was 4 (range 0–9). Patients over age 60 had significantly higher HCT-CI scores than younger patients (*p* = 0.04), as well as significantly more HCT-CI points related to cardiopulmonary co-morbidities (*p* = 0.04), (Supplementary Fig. 2). Across all patients, there was significant pulmonary dysfunction (moderate, 7 (23%); severe, 16 (52%)), most often manifested by low DLco and likely related to disease involvement for many. Idiopathic pneumonia syndrome (IPS) was diagnosed in two patients, including one patient in mid-February of 2020, before SARS-CoV2 testing was available. Intensive care unit-level support was required at least once for 15 (48%) patients, and 12 required mechanical ventilation. Five of these ventilated patients survived long-term with meaningful recovery and function, including a patient who also required extra-corporeal membrane oxygenation. Renal replacement therapy (RRT) was necessary in five patients, two of whom recovered from RRT needs. Sinusoidal obstructive syndrome occurred late (day +43) post-HCT in one patient, during recovery from SARS-CoV2 and arising concurrently with thrombotic microangiopathy (TMA) and who shortly thereafter developed grade II aGVHD and IPS. One patient had presumed inflammatory/immune neurotoxicity, requiring systemic steroids.

Transplant-associated (TA)-TMA occurred in four patients, with one patient having two discrete instances, with possible co-factors of sirolimus (*n* = 2), conditioning chemotherapy (*n* = 1), SARS-CoV-2 infection (*n* = 1), and SARS-CoV-2 vaccine (*n* = 1). Eculizumab was given to treat TA-TMA in three instances, to resolution in two (durations of 2 and 3.5 months of therapy) and until death in one; one had biopsy-proven TA-TMA but did not require therapy.

Protection from fatal infectious complications, including SARS-CoV2, was overall favorable, with infectious complications in the first 100-days shown in Table 4. There was intentional antibiotic stewardship to remove empiric, broad-spectrum antibacterial coverage after culture-negative neutropenic fevers deemed to be cell-infusion-associated cytokine release syndrome (CRS)-related fevers between day 0 and day +5 post-HCT in the setting of HLA-mismatched grafts. That said, bacteremia during aplasia was common (Table 4), and sepsis was the primary cause of early death for one patient and a contributor to volume overload and engraftment syndrome death in 2 patients (Table 3). Viral complications known to be common after PTCy-based HCT were observed. The overall day-180 Cul of human cytomegalovirus (CMV) infection requiring pre-emptive therapy was 48% (95% CI 30–65%), with similar rates among recipients of HLA-matched grafts compared to those of HLA-mismatched grafts. However, CMV disease was rare, occurring in only one patient at day +138 in the setting of systemic immunosuppression for cGVHD. BK viruria and viremia were frequent, detected in 84% and 71% of patients, respectively, on weekly monitoring post-HCT. Fifteen patients developed BK virus-associated cystitis/urethritis at a median of 32 days (range 15–137) post-HCT, which resolved after a median of 30 days (range 11–86). Despite the addition of hATG, this PTCy-based platform afforded good control of Epstein-Barr virus (EBV); there were no cases of EBV-PTLD, and most detection of EBV DNA in blood/plasma occurred during conditioning in the setting of EBV+ tumors (ENKTL) or AITL-associated EBV+ B-cell infiltrates (Table 4). Of 29 patients alive and post-HCT during the COVID-19 pandemic, four had COVID in the first 180 days post-HCT, eight had COVID between day +180 and 2-years post-HCT; six were hospitalized, two required intubation, and two developed a chronic supplemental oxygen requirement. Starting in 2021, SARS-CoV2 vaccination was encouraged as a high-priority vaccine to start after day +100. There were no deaths due to COVID, but unfortunately one death attributed to complications of the SARS-CoV2 vaccine.

**Table 4:** Infectious complications in the first 100-days post-HCT, by organ involved and pathogen

Pathogen by site	Recipients (*n* = 31)
Blood, *n* (%)	
CMV requiring pre-emptive therapy	11 (35%), 15 episodes
EBV DNAemia	13 (42%)
During conditioning only	10 (32%)
In setting of EBV+ PTCL or EBV+ B-cell infiltrate (AITL)	6 (19%)
Treated with anti-B-cell therapy^a^	1 (3%)
Bacteremia	12 (39%)
During aplasia	10 (32%)
Followed by fatal decompensation	3 (10%)
Urosepsis, in the setting of CBI	1 (3%)
In the setting of disease relapse and chemotherapy	1 (3%)
HHV6 viremia (asymptomatic, untreated)	17 (55%)
JCV viremia (asymptomatic, untreated)	9 (29%)
Adenoviremia (asymptomatic)^b^	1 (3%)
Toxoplasmosis^c^	1 (3%)
Viral upper respiratory infection, *n* (%)	3 (10%)
Pulmonary, *n* (%)	
Bacterial pneumonia	2 (6%)
SARS-CoV2	2 (6%)
Fungal pneumonia (confirmed *Aspergillus*, possible *Mucor*)	1 (3%)
Presumed *Pneumocystis jirovecii* pneumonia	1 (3%)
RSV	1 (3%)
Gastrointestinal, *n* (%)	
*Clostridioides difficile* colitis	2 (6%)
Norovirus	1 (3%)
*Giardia lamblia*	1 (3%)
Shiga-toxin *E. coli*	1 (3%)
Urinary, *n* (%)	
BKV-associated cystitis	13 (42%)
Bacterial urinary tract infection	6 (19%)
Adenoviruria (untreated)	3 (10%)

*CMV* human cytomegalovirus, *EBV* Epstein-Barr virus, *PTCL* peripheral T cell lymphoma, *AITL* angioimmunoblastic T cell lymphoma, *cGVHD* chronic graft versus host disease, *CBI* continuous bladder irrigation, *HHV6* human herpesvirus 6, *JCV* John Cunningham virus, *SARS-CoV2* severe acute respiratory syndrome coronavirus 2, *RSV* respiratory syncytial virus, *BKV* BK virus.

^a^Rituximab given for chronic GVHD, but agent choice motivated by rising EBV DNAemia.

^b^Resolved without treatment upon completing GVHD prophylaxis.

^c^Diagnosed concurrent with fatal disease progression.

### Exploratory cytokine profiling

CRS was observed in 62% of HLA-mismatched graft recipients between cell administration on day 0 and PTCy on days +3/+4, but did not exceed grade 2 and resolved by day +5 in all patients, requiring tocilizumab in four patients. On day +3, recipients of HLA-mismatched grafts had significantly higher levels of inflammatory cytokines compared to recipients of HLA-matched grafts, including interferon (IFN)-γ (*p* = 0.001), interleukin (IL)-5 (*p* = 0.01), IL-6 (*p* = 0.01), IL-10 (*p* = 0.01), CXCL9 (*p* = 0.04), and tumor necrosis factor (TNF)-α (*p* = 0.05), Supplementary Fig. 3.

Serial IL-8 levels between different subgroups are shown in Supplementary Fig. 4. Higher day +7 levels of IL-8 were observed in patients >60 years (*p* = 0.03), those who developed aGVHD (*p* = 0.01), and those with TRM before day +100 (*p* = 0.01). In addition to day +7 differences, patients who experienced early TRM had serially higher levels of IL-8 at day 0 (*p* = 0.008), day +5, and +14 (*p* = 0.01). Finally, higher IL-8 levels on day +5 were associated with increased need for additional immunosuppression beyond GVHD prophylaxis by day 100 (*p* = 0.003).

Noninfectious fevers were observed with engraftment in 10 patients, with engraftment syndrome in five patients. Engraftment syndrome was a factor in fatal respiratory failure in three patients. Patients with engraftment syndrome had significantly higher levels of day +14 IL-6 (*p* = 0.01). Overall, tocilizumab was given for engraftment syndrome in 2 patients, and for other manifestations thought to be IL-6-mediated in 4 additional patients. Compared to younger patients, patients >60 years had higher day +7 levels of CXCL9 (*p* = 0.05), with a tendency toward elevation in day +7 IFN-γ (*p* = 0.07). Emapalumab was given when clinically indicated, administered to one patient (P1) during graft instability, thought to be IFN-γ driven.

### Immune reconstitution

The kinetics of conditioning lymphodepletion, as well as recovery of CD3^+^ T-, CD8^+^ T-, CD4^+^ T-, NK-, and B-cell subsets, are shown in Supplemental Fig. 5, with no difference in the level of recovery at study timepoints by study arm, CMV-serostatus, or HLA-match. By day +100, 91% of surviving, engrafted patients had CD4^+^ T cells >50 cells/mcL, and 77% of surviving, engrafted patients had CD19^+^ B cells >25 cells/mcL, thresholds shown to be associated with lower TRM^30,31^. By day +180, 62% reached CD4^+^ T cells >200 cells/mcL, with 84% reaching this threshold at 1-year. Among recipients who completed the post-HCT series of conjugate and polysaccharide pneumococcal vaccines, protective anti-pneumococcal responses for ≥70% of the 23 serotypes was seen in 73% of patients.

### ATG levels and binding

We explored the pharmacokinetics and pharmacodynamics of distally-timed hATG in serially collected patient plasma specimens, both by quantifying total plasma levels and flow-cytometrically quantifying the binding of hATG in patient plasma to cell subsets. Total hATG levels in plasma declined during conditioning, with no differences in levels at each timepoint or in area under the curve by arm (Supplementary Fig. 6A) or by pre-HCT lymphocyte count (Supplementary Fig. 6B), with overall low levels for all patients at day 0, but with much heterogeneity. With regard to hATG binding, key immune and hematopoietic subsets did not have high levels of binding by hATG in patient plasma on day 0 (Table 5), consistent with our hypothesis that the distal timing would allow cytotoxic levels to decline by day 0, acting on host cells during conditioning and not on donor cells from day 0 and beyond (Supplementary Figs. 7 and 8). In general, we did not find that filgrastim during conditioning (mRIC arm, day −12, −8, and −4) increased hATG binding, as compared to the RIC arm, although higher binding at day −7, after day −8 filgrastim was seen for hematopoietic stem cells (HSCs), immature NK cells, and mature NK cells, but due to a drop in binding on the RIC arm more so than a rise in binding on the mRIC arm (Supplementary Figs. 7 and 8). An exception to this was relatively high binding on day 0 to B cells and myeloid-derived suppressor cells (MDSCs) (Table 5). There was significantly higher day 0 binding to HSCs, CD8^+^ TEMRA cells, immature and mature NK cells, and MDSCs on the mRIC arm, as compared to the RIC arm, as well as a single reversal of this pattern with higher binding to CD4^+^ TSCM cells on the RIC arm compared to the mRIC arm (Table 5). There was no significant relationship between the percentage of HSC binding on day 0 and time to engraftment or graft failure (GF) events.

**Table 5:** Binding of hATG in patient plasma on day 0 to healthy donor hematopoietic cell subsets

Day 0 binding by hATG in patient plasma (median % of parent)	RIC arm (*n* = 21)	mRIC arm (*n* = 10)	*p*-value
Hematopoietic stem cells (CD15^−^CD14^−^CD34^+^)	1.8%	10.1%	0.0002
B cells (CD15^−^CD14^−^CD34^−^CD19^+^)	83.7%	81.2%	NS
CD4 T cells (CD15^−^CD14^−^CD34^−^CD19^−^CD3^+^CD4^+^)	3.5%	4.3%	NS
CD4 TEMRA (CD25^−^Foxp3^−^CD45RA^+^CCR7^−^)	13.2%	14.3%	NS
CD4 effector memory (CD25^−^Foxp3^−^CD45RA^−^CCR7^−^)	3.9%	5.3%	NS
CD4 central memory (CD25^−^Foxp3^−^CD45RA^−^CCR7^+^)	6.1%	6.5%	NS
CD4 true naïve (CD25^−^Foxp3^−^CD45RA^+^CCR7^+^CD95^−^)	3.2%	10.4%	NS
CD4 TSCM (CD25^−^Foxp3^−^CD45RA^+^CCR7^+^CD95^+^)	24.1%	18.2%	0.03
CD4^+^ regulatory T cells (CD25^+^Foxp3^+^)	9.0%	19.8%	NS
CD8 T cells (CD15^−^CD14^−^CD34^−^CD19^−^CD3^+^CD8^+^)	2.5%	4.5%	NS
CD8 TEMRA (CD45RA^+^CCR7^−^)	1.5%	4.4%	0.004
CD8 effector memory (CD45RA^−^CCR7^−^)	2.7%	6.3%	NS
CD8 central memory (CD45RA^−^CCR7^+^)	7.2%	11.0%	NS
CD8 true naïve (CD45RA^+^CCR7^+^CD95^−^)	1.4%	3.5%	NS
CD8 TSCM (CD45RA^+^CCR7^+^CD95^+^)	14.4%	14.4%	NS
Immature NK cells (CD15^−^CD14^−^CD34^−^CD19^−^CD3^−^CD56^+bright^CD16^−^)	10.0%	40.9%	0.0001
Mature NK cells (CD15^−^CD14^−^CD34^−^CD19^−^CD3^−^CD56^+int/low^CD16^+^)	2.1%	16.7%	0.00002
Myeloid-derived suppressor cells (CD15^−^CD14^+^HLA-DR^−^)	61.2%	73.8%	0.001

Mann-Whitney tests (two-sided, without adjustments) are performed to compare binding between the two arms.

*hATG* horse anti-thymocyte globulin, *RIC* reduced-intensity conditioning, *mRIC* modified RIC, *NS* not significant, *TEMRA* terminally differentiated effector memory T-cells, *TSCM* stem cell memory T-cells, *NK* natural killer.

## Discussion

The HCT platform in this study is a unique regimen, with the selection, timing, and duration of agents tailored to the needs of patients with PTCL undergoing HCT. The approach is reduced-intensity, as myeloablative levels of conditioning are not needed in mature lymphoid malignancies, and add barriers to HCT eligibility and greater toxicity. To augment host lymphodepletion and perhaps anti-T-cell tumor activity, horse ATG was used. In contrast to standard uses of rabbit ATG (rATG) in HCT, timed proximally enough to serve a dual purpose of (1) host conditioning and (2) in vivo T-cell depletion for GVHD prophylaxis, the intent in this study was to use hATG, which has a shorter half-life than rATG, and time the hATG distally enough to act only on the host, hypothesizing that distally timing would result in negligible levels at day 0, avoiding in vivo T-cell depletion to result in a pure, PTCy-based GVHD prophylaxis approach and foster robust IR and GVT. This hypothesis was supported by our exploratory analyses of total hATG levels, hATG binding to key hematopoietic and immune cell subsets, and IR kinetics. Unlike investigations into model-based precision dosing of rATG, where the intent is to personalize the dose of rATG to result in levels beyond day 0 that effectively prevent GVHD while also optimize IR and lessen TRM^32^, this study’s sole intent was to have negligible levels of hATG by day 0. However, with the study of more patients with distally-timed hATG, we will further investigate if dosing (or timing) based on pre-HCT factors might improve outcomes.

The outcomes of HCT for PTCL have recently been reviewed by others, where data are largely retrospective and heterogeneous in disease, patient, graft, and HCT platform characteristics, but overall show a 3-year PFS of ~50% and 3-year OS of ~60% for patients largely in remission prior to HCT^33,34^. A registry study showed RIC HCT recipients (*n* = 45) to have an estimated 3-year PFS of 33%, with 3-year OS of 52%, 1-year TRM of 27%, and 3-year relapse of 40%^22^. Subsequent to the 2022 review^33^, Johns Hopkins reported their retrospective outcomes of non-myeloablative HCT for PTCL in remission (*n* = 65), where all patients received PTCy-based GVHD prophylaxis, many received HLA-haploidentical grafts, many histologies were included, and adult patients of all ages, including those older than age 60, were represented^35^. In that study, 2-year PFS of 49%, 2-year OS of 55%, 1-year relapse of 25% and 1-year TRM of 12%^35^. Similar to our study, grade III–IV aGVHD was low (3% in their study, 0% in our study)^35^. Recipients of PBSC grafts (*n* = 19) had remarkably better 2-year PFS and OS of 79% and 84%, respectively, although significantly shorter median follow-up compared to recipients of marrow grafts^35^. In a German study that compared PTCL HCT outcome to those for aggressive, relapsed/refractory B-NHLs, GVT was noted to be more potent in PTCL vs B-NHL, with a benefit seen in those with extensive cGVHD^36^. Prospective studies are few and differ in many ways from our cohort^27,37^, although the power of the GVT effect in PTCL has been noted with clear examples for decades^36,37^. In a similarly-sized (*n* = 28), recently fully-accrued prospective study of patients in at least PR, 3-year PFS and OS were 41% and 54%, respectively, with relapse of 33% at 3 years and 1-year TRM of 21%^27^.

Our outcomes reinforce the possibility of cure in otherwise incurable PTCLs, the need for reduced-intensity, lower-toxicity approaches, and the role of HCT/GVT immunity for these diseases, while also highlighting new insights and opportunities to improve. While limited by TRM, the favorable relapse outcomes among survivors in our study for our patients suggest that myeloablative conditioning may not be important for PTCL and that RIC is sufficient, thus opening up HCT accessibility to a broader pool of patients. As our study was ongoing, a retrospective report of PTCL HCT outcomes from Johns Hopkins noted superior outcomes with PBSC grafts, with a recommendation that PBSC is the preferred graft source in PTCL^35^. Despite the higher risk of cGVHD, PBSC grafts were chosen for this study to afford faster engraftment, better IR, and GVT, and our outcomes demonstrate that risk/benefit trade-off of using PBSCs. While it is the standard in the field to require some element of remission to proceed to HCT for PTCL, this trial did not require remission to be eligible, and the majority of patients indeed had chemorefractory, actively progressing disease pre-HCT. This is the most remarkable aspect of our study population, setting it apart from most existing data in these diseases. The absence of severe aGVHD, coupled with the examples of post-HCT remission over the first few post-HCT months despite active pre-HCT disease, suggests that GVT and GVHD may be dissociated. Even though GVT from the initial HCT graft/cell infusion was demonstrated in several patients, this does not reliably extend to subsequent cell infusions, such as DLI, as we have shown overall low efficacy of DLI after PTCy-based HCT^29^, particularly against a galloping pace of PTCL progression. As a cautionary tale, treatment of GVHD was followed by relapse/progression in some of our patients, a reminder that these lymphomas continue to lurk subclinically, controlled if monitored closely and IR/GVT is cultivated. As patients were transplanted on this trial, the importance of limiting systemic immunosuppression became more apparent and a practice developed where any need for systemic immunosuppression was coupled with disease re-assessment before starting immunosuppression (when possible) and disease monitoring while on immunosuppression.

While the PFS, relapse, OS, and severe aGVHD rates are overall promising, the high rates of early TRM in older patients warrant further consideration to improve the platform. The cytokine elevations in IL-8 and the IFN-γ pathway in older patients on day +7, after PTCy abrogated clinical CRS, are of note as a direction for early intervention to decrease peri-engraftment cardiopulmonary toxicities, particularly in recipients of HLA-mismatched grafts. Work by others has demonstrated increased risk of aGVHD in those with high IL-8 at day +14^38^. IL-8 can drive neutrophil extracellular trap (NET) release, in turn causing endothelial injury, particularly around neutrophil engraftment when NET release is high^39^. Investigation into post-HCT cytokine levels as early prognostic markers will continue on this and parallel studies.

Our study results support the power of GVT to overcome chemorefractory disease, cultivated within an allogeneic HCT approach that affords abbreviated GVHD prophylaxis while avoiding severe aGVHD and allowing for early, robust IR. Remission should not be required to proceed to HCT for PTCL, particularly if remission seems improbable and the patient is young, as further chemotherapy will likely introduce added toxicity, thus increasing TRM risk related to comorbidities, while delaying allogeneic HCT. Early CNS screening may minimize additional delays and positive CSF, while not ideal, might not be an absolute barrier to proceed to HCT. Use of HLA-mismatched donors, related and unrelated, afforded donor options for an ethnically diverse referral base and study cohort, yielding a HCT cohort that approximates the ethnic/racial diversity in PTCL incidence rates^40^. We continue to prospectively study allogeneic HCT for PTCL, including ongoing study of adult T-cell leukemia/lymphoma (ATL)-specific approaches with promising early results. The future of allogeneic HCT for PTCLs hinges on further harnessing GVT without undue toxicities, particularly when transplanting patients with active disease that may make post-HCT inflammatory responses and organ dysfunction more likely.

## Methods

### Study design

This is an institutional review board-approved, single-center, prospective clinical trial (NCT03922724, clinicaltrials.gov) conducted at the National Institutes of Health Clinical Center from April 18, 2019, to present, with an enrollment pause from August 23, 2022–December 19, 2023, for data analysis. The clinical data from all patients enrolled and evaluable on the RIC or mRIC arm through August 23, 2022, are included. All patients provided written informed consent prior to study enrollment. Eligible patients were age ≥12 years with mature neoplasms of T- or NK-cell origin that were either relapsed/refractory, or of a risk category based on prognostic index for T-cell lymphoma score or clinical practice guidelines^15,16,41^ wherein upfront HCT in first-remission is considered an appropriate therapeutic option. Risk was assessed using the HCT-CI^42^. Ethnicity/race was self-reported.

### Transplantation platform

Patients were transplanted on the RIC arm, followed by a mRIC arm upon full accrual to the RIC arm. The HCT platform is outlined in Table 6 but in brief, the conditioning was radiation-free and was comprised of horse ATG on days −14 and −13, pentostatin on days −11 and −7, low-dose, hyperfractionated cyclophosphamide on days −11 to −4, and pharmacokinetically-dosed busulfan on days −3 and −2. Horse ATG was timed 2-weeks prior to day 0, with the hypothesis that the distal timing would act only on the host for conditioning and T-cell tumor control, reaching negligible levels by day 0 and avoiding in vivo donor T-cell depletion post-HCT. The mRIC arm modified the RIC approach with the addition of filgrastim on days −12, −8, and −4, with the hypothesis that filgrastim may augment the clearance of hATG-bound cells^43^ during conditioning. ATL patients were eligible on the RIC arm but excluded on the mRIC (ATL patients were transplanted instead on an ATL-RIC arm that was added in 2021 to translate emerging ATL-specific work from bench to bedside). GVHD prophylaxis was high-dose PTCy-based, with adjunct immunosuppressive agents ending by day +60; the hATG, given its distal-timing, was not considered part of the GVHD prophylaxis approach.

**Table 6:** HCT platform and supportive care measures

Day	RIC	mRIC
Prior to day −14	Busulfan test dose −0.8 mg/kg IV to calculate AUC^a^	
−14 and −13	Equine anti-thymocyte globulin 40 mg/kg/day IV	
−14 through −7	Prednisone taper: days −14 through −12: 1 mg/kg/day; days −11 and −10: 0.75 mg/kg/day; days −9 and −8: 0.50 mg/kg/day; day −7: 0.25 mg/kg/day^b^	
−11 and −7	Pentostatin 4 mg/m^2^/day IV	
−11 through −4	Cyclophosphamide 5 mg/kg/day orally or IV, dosage cap of 400 mg/day	
**−12, −8, and −4**	**N/A**	**Filgrastim 5 mcg/kg/day subcutaneously**
−3 and −2	Busulfan pharmacokinetically-dosed, with targeted 24-h systemic exposure of 18.9 mg × h/L (range 14.8–23.0 mg × h/L/24-h based on test dose AUC or real-time pharmacokinetics on day −3 to inform day −2 dosing^a^	
0	T-cell replete PBSC graft: target of at least 5 × 10^6^ CD34^+^cells/kg recipient IBW; usually capped at 10 × 10^6^ CD34^+^cells/kg IBW. Fresh grafts were preferred, but cryopreservation was utilized during the SARS-CoV2 pandemic, particularly for unrelated grafts, per global guidelines.	
+3 and +4	Cyclophosphamide 50 mg/kg/day IV^c^ with mesna 50 mg/kg/day IV^c^ beginning 60–72 h after graft infused	
+5	Sirolimus loading dose, goal trough of 5–12 ng/mL	
	Mycophenolate mofetil 15 mg/kg orally or IV three times daily; maximum 1000 mg/dose	
	If somatic (or germline) mutation in the Akt/mTOR pathway: tacrolimus INSTEAD of sirolimus: IV continuous infusion or orally twice daily (*n* = 1 RIC arm patient)	
+6	Sirolimus maintenance dose: goal trough of 5–12 ng/mL; if tacrolimus, goal trough of 5–10 ng/mL	
+25	Discontinue MMF after the last dose	
+60	Discontinue sirolimus or tacrolimus after the last dose, no taper	

Day	Treatment or supportive care measure and indication	Dosing/other specifics
Prior to day −14	Leuprolide for ovarian suppression	Premenopausal females
Prior to day −14	Ivermectin	If from area endemic for strongyloides; 200 mcg/kg orally × 1–2 doses
−4 through −1	Clonazepam for seizure prophylaxis	0.5 mg orally twice daily
−4 through −1	Levetiracetam for seizure prophylaxis	500 mg orally twice daily
−11 through +100	Ursodiol for hepatic protection	<90 kg: 300 mg orally twice daily; ≥90 kg: 300 mg each morning and 600 mg each evening, orally
−11 through +100 or longer	Antifungal prophylaxis	Agent chosen as indicated and tolerated by organ function and drug interactions
−11 through +2 years	Alphaherpesvirus prophylaxis: acyclovir	800 mg orally twice daily
+5 through +100	CMV prophylaxis	CMV seropositive recipients: letermovir 480 mg or appropriate dose reduction, orally once daily
Upon count recovery through +1 year	PJP ± toxoplasmosis prophylaxis: trimethoprim-sulfamethoxazole or alternative	
0 until prophylaxis resumed	Twice weekly whole blood qualitative PCR for toxoplasmosis in seropositive recipients	
0 through +5	Avoidance of corticosteroids; hydrocortisone permitted for adrenal insufficiency	
+3 through +5	IV or oral hydration and voiding for uroprotection	Goal urine output of 150–200 cc/h and voiding every 2 h for 48 h, from the start of PTCy
+5 until ANC ≥ 1000/µL for 3 days	Filgrastim	RIC arm only: 5 mcg/kg/day subcutaneously
+100 onward	Post-transplantation immunizations	Prioritization of SARS-CoV2 vaccine at day +100; other vaccines started at day +100 if early IR, or day +180 if standard IR/schedule

Differences between the RIC and mRIC arm are bolded. Weight-based dosing utilizes actual body weight unless otherwise noted.

*IV* intravenous, *AUC* area under the curve, *IBW* ideal body weight, *PJP* Pneumocystis jiroveci pneumonia, *PCR* polymerase chain reaction, *CMV* human cytomegalovirus, *ANC* absolute neutrophil count, *PTCy* posttransplantation cyclophosphamide, *IR* immune reconstitution.

^a^Alternatives to prednisone were permitted, such as dexamethasone in those with CNS disease. Additional steroids (up through day −1) and/or antihistamines were given for serum sickness or infusion-related reactions, as clinically indicated.

^b^Busulfan test dose was based on ideal body weight or actual body weight (ABW), whichever was lower, unless the recipient was > 120% of IBW, in which case adjusted ideal body weight (IBW + 25% of the difference between IBW and ABW) was used. In 2021, in-house, real-time busulfan PK levels were available and used in lieu of the busulfan test dose when possible.

^c^Dosed according to IBW, unless the recipient weighed less than IBW, in which case ABW was used.

### Endpoints

The primary endpoint was PFS at 1-year post-HCT, powered to be analyzed separately by arm. The study was statistically designed as a Simon 2-stage to have 80% power in the RIC arm to determine whether there is a difference between a 45% 1-year PFS probability and an improved 70% 1-year PFS probability, with a one sided 0.10 alpha level test, using the method of Brookmeyer and Crowley^44^; the Simon 2-stage design was not repeated for the subsequent mRIC arm given progression to stage 2 in the RIC arm.

Other endpoints were analyzed across all patients or in exploratory subgroups. Secondary endpoints included OS and the CuI of aGVHD, cGVHD, GF, progression/relapse, infectious complications, and TRM. Acute and cGVHD were diagnosed and graded using standard criteria^45,46^. Neutrophil recovery was defined as the first of three days when a post-nadir absolute neutrophil count reached ≥500/mm^3^. Engraftment syndrome was defined using Spitzer criteria^47^. Primary GF was defined as <5% donor myeloid chimerism in blood and/or bone marrow on all evaluations up to and including day +60, in the absence of a recurrent marrow malignancy. Secondary GF was defined as having achieved neutrophil recovery and initial blood or marrow donor myeloid chimerism ≥5%, declining to <5% on subsequent measurements. The disease status of the patient’s PTCL pre- and post- HCT was evaluated with testing and imaging as appropriate for the PTCL subtype and manifestations. CRS was graded per the ASTCT grading scale^48^; tocilizumab was used as clinically indicated. Additional clinical monitoring is detailed in the Supplementary Information.

### Correlative studies

Methodology related to ATG quantification in plasma, flow cytometric ATG binding studies, immune subset analyses, and cytokine profiling are in the Supplementary Information.

### Statistical analysis

Data were locked for analysis on July 18, 2024. Survival endpoint probabilities were estimated using the Kaplan–Meier method with 95% CIs. CuI of TRM, GVHD, CMV infection, and GF were estimated by competing-risk analysis using Gray’s method^49^. Death was a competing risk for all analyses except TRM; PTCL relapse/progression was a competing risk for TRM. As many patients had active disease pre-HCT and disease status was not fully assessed at times of critical illness/decompensation in the early post-HCT period, the data coding approach was to consider these deaths as TRM, even though some of these patients may have also had active/progressive disease at the time of death. GF was considered a competing risk for CMV infection, aGVHD, and cGVHD. Chronic GVHD served as an additional competing risk for aGVHD. Data were analyzed with R Statistical Software (v4.3.2; R Core Team, 10-31-2023, Vienna, Austria) and Prism (v10.3.1, GraphPad Software, La Jolla, CA).

### Clinical monitoring

Patients were evaluated by a study team clinical provider daily as inpatients from the start of conditioning to discharge post-engraftment, followed by at least weekly through day +100, then at least at study timepoints of day +180, 1 year, 1.5 years, then yearly from years 2 through 5, and as clinically indicated.

Infection monitoring performed included weekly PCR-based studies through day +100 of EBV, CMV, human herpesvirus 6 (HHV6), human adenovirus (AdV), BK polyomavirus (BKV), JC virus (JCV), and toxoplasmosis in blood, and weekly BKV, JCV, and AdV in urine. CMV monitoring, prophylaxis, and preemptive treatment for clinically significant CMV viremia were performed per institutional guidelines.

### Plasma hATG concentration assays

ELISA to determine hATG total levels in plasma at serial timepoints was performed at serial timepoints: day −14 (pre-hATG), −10, −7, −3, 0, +3, +5, +7, and +14. ELISA plates (Cat#439454. Nunc, Roskilde, Denmark) were coated with 100 μL/well rabbit anti-horse IgG (Fab’2) (LS-C60476, 2 mg/ml. 1:400 for coating. Lot#177112. LifeSpan BioSciences, Seattle, WA; validated for ELISA per manufacturer) in coating buffer (carbonate-bicarbonate buffer, C3041-50CAP. Sigma) overnight at 4 °C. Coated plate was washed twice with PBS containing 0.05% tween-20 (PBST). To prevent nonspecific binding, the plates were blocked 1% bovine serum albumin/phosphate-buffered solution (BSA/PBS) for 1 h at room temperature (RT). Plates were washed 3 times with PBST. Fifty microliters plasma samples diluted 1:1000 with 1% BSA/PBS were added to each well in duplicate and incubated at RT for 1 h. Series diluted hATG (Atgam, Upjohn, Kalamazoo, MI) were used as standards (500, 250, 125, 62.5, 31.2, 15.6, 7.8, and 0 ng/ml). Plates were washed 5 times with PBST, and then 100 μL of diluted biotin-conjugated goat anti-horse IgG (Fab’2) antibody at 1:100,000 with 1% BSA/PBS (Cat#LS-C60467, LifeSpan BioSciences, Lot#177110; validated for ELISA per manufacturer) were added to all wells and incubated for 1 h). Plates were washed 5 times with PBST, then one hundred µl/well avidin-horseradish peroxidase (1:2000 diluted with 1% BSA/PBS) (Cat#18-44100-51. eBioscience, San Diego, CA) were added and incubated for 30 min. Plates were washed for 7 times followed by the addition of 100 μl Tetramethylbenzidine (TMB, Cat#00-4201-56. eBioscience) substrate solution and incubated until color development. One hundred microliters 2N H_2_SO_4_ were added to stop the reaction, and the optical density (O.D.) at 450 nm measured with a microtiter plate reader (Victor 3, PerkinElmer, Waltham, MA).

- Coating: 27.5 μl rabbit anti-horse IgG (Fab’2) (LS-C60476) + 11 ml coating buffer for 1 plate, 100 μl/well
- Standards: 2 μl ATG stock (50 mg /ml) + 1 ml 1%BSA/PBS (1:500 = 100,000 ng /ml), then take 10 μl diluted ATG + 1 ml 1%BSA/PBS (1:100 = 1000 ng /ml). S1-S7: Serial 2-fold dilution, S8 = 1%BSA/PBS
- Plasma samples: 1:1000 dilution with +1 ml 1%BSA/PBS
- Detection Ab: 10 μl biotin-conjugated goat anti-horse IgG (Fab’2) antibody (Cat#LS-C60467) + 1 ml 1% BSA/PBS (1:100 dilution); then 12 μl diluted detection Ab + 12 ml 1% BSA/PBS (20 ng/ml, final dilution = 100,000)
- Enzyme: 6 μl avidin-horseradish peroxidase + 12 ml 1%BSA/PBS (1:2000 dilution)

### ATG binding assays

An unmanipulated stem cell product from a single healthy donor was used for hATG binding assessments of all patient plasma samples at serial timepoints, with a focus on the day 0 timepoint to assess binding (as a surrogate for cytotoxicity) of residual hATG in patient plasma on HCT day and administration of donor cells. Sequential staining occurred as follows: LIVE/DEAD fixable Aqua dead cell stain kit (Invitrogen; cat #L34966) for 30 minutes (min) at room temperature (RT), Fc block (Human TruStain FcX; Biolegend; cat #422302) for 10 min at RT, incubation of the cells with 10 μl of the respective patient plasma and an additional 50 µl of PBS on ice for 30 min, incubation with fluorescein isothiocyanate (FITC)-conjugated polyclonal goat anti-horse IgG (Fab’2) secondary antibody (Lifespan Biosciences; cat. #LS-C60461-2, validated for flow cytometry per manufacturer) for 20 min at RT, extracellular markers for 20 min at RT, fixation and permeabilization (eBioscience FOXP3/Transcription Factor Staining Buffer Set; Invitrogen; cat #00-5523-00) for 30 min at RT, and intracellular marker staining for 20 min at RT. Wash steps occurred between each of the above sequential staining steps. Antibodies used for staining, all validated for flow cytometry use per manufacturer, with amount per 100 μl test included, include: BUV395 mouse anti-human HLA-DR (clone G46-6; BD Biosciences; cat #564040, 0.5 μL), BUV737 mouse anti-human CD4 (clone SK3; BD Biosciences; cat #612748, 2uL), BUV805 mouse anti-human CD14 (clone M5E2; BD Biosciences; cat #612902, 2.5 μL), BV605 mouse anti-human CD56 (clone 5.1H11; Biolegend; cat #362538, 5 μL), BV650 mouse anti-human CD15 (clone HI98; BD Biosciences; cat #564232, 0.5 μL), BV711 mouse anti-human CD197 (clone G043H7; Biolegend; cat #353228, 5 μL), BV786 mouse anti-human CD19 (clone SJ25C1; BD Biosciences; cat #563325, 0.5 μL), PerCP/Cy5.5 mouse anti-human CD8 (clone SK1; Biolegend; cat #344710, 1 μL), PE mouse anti-human CD16 (clone 3G8; Biolegend; cat #302008, 0.5 μL), PE-CF594 mouse anti-human CD95 (clone DX2; BD Biosciences; cat #562395, 2 μL), PE/Cy7 mouse anti-human CD25 (clone BC96; Invitrogen; cat #25-0259-42, 1.5 μL), APC mouse anti-human CD34 (clone 581; BD Biosciences; cat #555824, 10 μL), AF700 mouse anti-human CD3 (clone UCHT1; Biolegend; cat #300424, 0.5 μL), APC/Cy7 mouse anti-human CD45RA (clone HI100; Biolegend; cat #304128, 1.5 μL), and eFluor 450 rat anti-human Foxp3 (clone PCH101; Invitrogen; cat #48-4776-42, 5 μL). Data were acquired on a BD Fortessa flow cytometer and analyzed using FCS Express. Gating strategy is shown in Supplementary Fig. 9.

### Immune and cytokine profiling

IR was assessed quantitatively by flow cytometry to enumerate T- (CD3^+^, CD4^+^, CD8^+^), B-, and NK-cell subsets weekly through day +100 and at subsequent study timepoints. Flow cytometry was used to analyze frequencies of immune cells on days from purified peripheral blood mononuclear cells (PBMCs). The following antibodies were used to determine frequencies of T cells, B cells, NK cells, and monocytes, all validated by the manufacturers: FITC mouse anti-human CD45 (Biolegend; cat #304006), PE mouse anti-human CD56 (BD; cat #555516), PerCP/Cy5.5 mouse anti-human CD16 (Biolegend; cat #302028), PECy7 mouse anti-human CD14 (Ebioscience; cat #25-0149-42), APC mouse anti-human CD19 (Biolegend; cat #302212), Alexa Fluor700 mouse anti-human CD3 (BD; cat #557943), BV410 mouse anti-human CD4 (Biolegend; cat #300518), and), eFluor 450 mouse anti-human CD8 (Ebiosciences; cat #48-0088-42). The stained cells were run on a Beckman Coulter CytoFLEX LX cytometer, and the data were analyzed using FCS Express. The following antibodies were used to stain purified PBMCs to sort CD4^+^CD3^+^, CD8^+^CD3^+^, CD56^+^CD3^−^,CD19^+^, and CD14^+^ cells for chimerism studies: FITC mouse anti-human CD45 (Biolegend; cat #304006), PE mouse anti-human CD56 (Miltenyi Biotec; cat #130-113-312), PECy7 mouse anti-human CD14 (eBioscience; cat #25-0149-42), APC mouse anti-human CD19 (Biolegend; cat #302212), Alexa Fluor700 mouse anti-human CD3 (BD; cat #557943), eFluor 450 mouse anti-human CD8 (eBioscience; cat #48-0088-42), BV410 mouse anti-human CD4 (Biolegend; cat #317444). Each patient sample for each timepoint was used as a biological replicate for flow cytometry. No technical replicates were performed for patient samples for flow cytometry. However, a different aliquot of the same healthy donor cells was used with each batch on each day, and different aliquots of the same healthy donor and patient sample plasma were also used on each day as controls.

Cytokine profiling of plasma levels of IFN-γ, TNF-α, CXCL9, IL-5, IL-6, IL-8, IL-10, and IL-13 was performed serially at days -14, 0, +3 (pre-PTCy), +5, +7, +14, and +28 using U-PLEX Human MIG Assay (Mesoscale, Rockville, MD) as per the manufacturer’s instructions. IL-6 levels obtained within 7-days post-tocilizumab were excluded from analyses.

### Reporting summary

Further information on research design is available in the Nature Portfolio Reporting Summary linked to this article.

## Supplementary information

Supplementary Information

Reporting Summary

Transparent Peer Review File

## Source data

Source Data

## Data Availability

The raw Source data generated in this study are provided in the Source data file. Source data are provided with this paper.
