# Supplementary material for: Allogeneic hematopoietic cell transplantation in mature T- or NK-lymphomas: a phase II clinical trial
Source: Nat Commun. 2026 Apr 23;17:5606. doi: 10.1038/s41467-026-71461-5 (PMC13316028; doi:10.1038/s41467-026-71461-5)
Supplement: Supplementary file 1 — Supplementary Information [file 41467_2026_71461_MOESM1_ESM.pdf]

SUPPLEMENTARY INFORMATION

Supplemental Table 1. Details of individual patient and donor demographics, graft dose, and outcomes.

Patient	Disease	Recipient Age (years) at HCT, Sex	HCT-CI	*Age HCT-CI	Donor Source, Age (years), Sex	Year of HCT	Graft details	Graft Failure	Subsequent Cell Infusion (indication)	Acute GVHD, maximum grade	Chronic GVHD (maximum severity)	Follow-Up
P1	ALK- ALCL	13, M	4	4	Haplo, 18, M	2021		Yes	DLI D+11 (split chimerism)	No	No	Died D+79<br>Cardiac arrest, during engraftment, off study after 2nd HCT (had graft failure on study)
P2	ALK- ALCL	38, F	6	6	Haplo, 35, F	2020		No	No	No	Mouth, skin, eyes, liver (severe)	A&W, off IS, +4 years
P3	ALK - ALCL	37, F	3	3	Haplo, 41, F	2020		No	No	No	No	A&W, off IS, +4.5 years
P4	ALK- ALCL	57, M	7	8	Haplo, 21, F	2020	F->M	No	No	Grade 2, skin	No	Relapse D+224. Treated with brentuximab and XRT now in CR. A&W, off IS, +4 years
P5	ALK- ALCL	37, M	7	7	mMUD, 31, F	2020	F->M, Cryo	No	No	No	No	Died D+207<br>SARS-CoV2 vaccine-related lymphocytic interstitial pneumonitis (reported to VAERS)
P6	ENKTL	37, F	4	4	Haplo, 28, F	2021		No	No	No	Lung, eyes (severe)	Alive, on IS, +3 years
P7	ENKTL	35, M	4	4	Haplo, 15, M	2022		No	No	No	No	A&W, off IS, +2.5 years
P8	ENKTL	22, M	3	3	Haplo, 30, M	2019		No	No	No	No	A&W, off IS, +5 years
P9	ENKTL	30, M	2	2	Haplo, 38, M	2020	Cryo	Yes	2nd HCT	No	No	Alive, censored at GF
P10	ENKTL	44, M	3	3	MSD, 36, M	2020		No	DLI D+260 (PD)	Grade 0, liver (unstageable)	No	Died D+309<br>Disease progression, occurring in the setting of treatment for acute GVHD
P11	MEITL	46, F	7	8	MUD, 23, F	2020		No	No	No	Skin (severe)	Alive, on IS for cGVHD, +3.5 years
P12	CTCL	27, F	3	3	Haplo, 31, F	2019	DSA+	Yes	DLI D+133 (relapse, HSV)	No	No	Alive, censored at GF
P13	CTCL	58, F	5	6	Haplo, 20, M	2021		No	No	No	No	Alive, off IS, +3.5 years
P14	CTCL	38, F	3	3	Haplo, 37, F	2019		No	No	Grade 1, skin	Oral, GI, genital, Skin, lungs (severe)	Alive, PD at D+120 treated with romidepsin + TSEB, off IS, +4.5 years.
P15	CTCL	29, M	0	0	Haplo, 32, M	2022		No	No	Grade 2, skin + GI	Skin, fascia, liver (severe)	Alive, on IS for cGVHD, +2 years
P16	PTCL-NOS	54, M	4	5	Haplo, 23, M	2020		No	No	No	No	Died D+663<br>Cardiac arrest while performing iADLs, with medical co-morbidities of DM2, HTN, HLD
P17	PTCL-NOS	71, M	7	8	Haplo, 41, M	2022	Cryo	No	No	No	No	Died D+10 of sepsis.
P18	PTCL-NOS	37, M	0	0	MSD, 30, F	2019	F->M	No	No	No	Mouth, genitals, eyes, liver, skin, lungs (severe)	A&W, on IS for AIH (not cGVHD), +4.5 years
P19	PTCL-NOS	62, F	4	5	MUD, 25, M	2020	Cryo	No	No	Grade 2, skin	No	A&W, off IS, +4years
P20	PTCL-NOS	67, F	2	3	Haplo, 37, F	2022		No	No	No	No	Died D+36, Engraftment syndrome, polymicrobial sepsis requiring volume resuscitation
P21	PTCL-NOS	61, M	6	7	MUD, 38, F	2021	F->M, Cryo	No	No	No	Ocular, oral, genital, and skin, liver (severe)	Alive, on IS for cGVHD, +2.5 years.
P22	AITL	61, F	7	8	Haplo, 31, F	2021		No	No	No	No	Died D+17<br>Brain death, after witnessed PEA arrest with inability to secure advanced airway
P23	AITL	61, F	3	4	MUD, 23, M	2021	Cryo	No	No	No	No	A&W, off IS, +3 years
P24	AITL	63, F	9	10	MUD, 28, M	2021	Cryo	No	No	No	No	A&W, off IS, +2.5 years
P25	AITL	59, F	3	4	MUD, 23, M	2022	Cryo	No	No	No	No	A&W, off IS, +2.5 years
P26	AITL	65, F	6	7	Haplo, 40, M	2022		No	No	No	No	Died D+20 Engraftment syndrome, polymicrobial sepsis requiring volume resuscitation
P27	AITL	64, M	7	8	Haplo, 28, M	2019		No	No	Grade 2, skin + GI	No	Died D+260<br>Sepsis (*Staphylococcus aureus)*, on outpatient parental nutrition due to malnutrition after gut GVHD and infectious enterocolitis
P28	AITL	51, F	4	5	mMUD, 28, F	2021	Cryo	No	CD34+ boost D+128 (PGF)	No	Skin, eye, mouth, genital (severe)	A&W, off IS, + 3 years
P29	ATL	51, F	3	4	MSD, 53, M	2019		No	No	Grade 2, GI	No	Died D+53 Respiratory failure, due to post-engraftment, non-infectious lung process related to lipoid and organizing pneumonia
P30	ATL	46, M	3	4	Haplo, 40, M	2020		No	DLI D+39 (PD)	No	No	Died D+50<br>Disease progression
P31	HSGDTCL	34, M	7	7	Haplo, 33, M	2020		No	DLI D+79 (PD)	No	No	Died D+205<br>Disease progression

Abbreviations: HCT, hematopoietic cell transplantation; HCT-CI, HCT-Comorbidity Index; TNC, total nucleated cell; IBW, ideal body weight; GI, gastrointestinal; GVHD, graft-versus-host disease; F, female; M, male; Haplo, HLA-haploidentical; MUD, 10/10 matched unrelated donor; MRD, matched sibling donor; mMUD, mismatched unrelated donor, CMV, cytomegalovirus; DLI, donor lymphocyte infusion; A&W, alive and well; IS, immunosuppression; EOD, evidence of disease; TRM, transplant-related mortality; PD, progressive disease; PEA, pulseless electrcical activity; PGF, poor graft function; AIH, autoimmune hepatitis; TSEB, total skin electron beam therapy; cryo, cryopreserved; iADL, instrumental activities of daily living; DSA, donor specific antibodies

* Sorror ML, Storb RF, Sandmaier BM, et al. Comorbidity-age index: a clinical measure of biologic age before allogeneic hematopoietic cell transplantation. J Clin Oncol. 2014;32(29):3249-3256

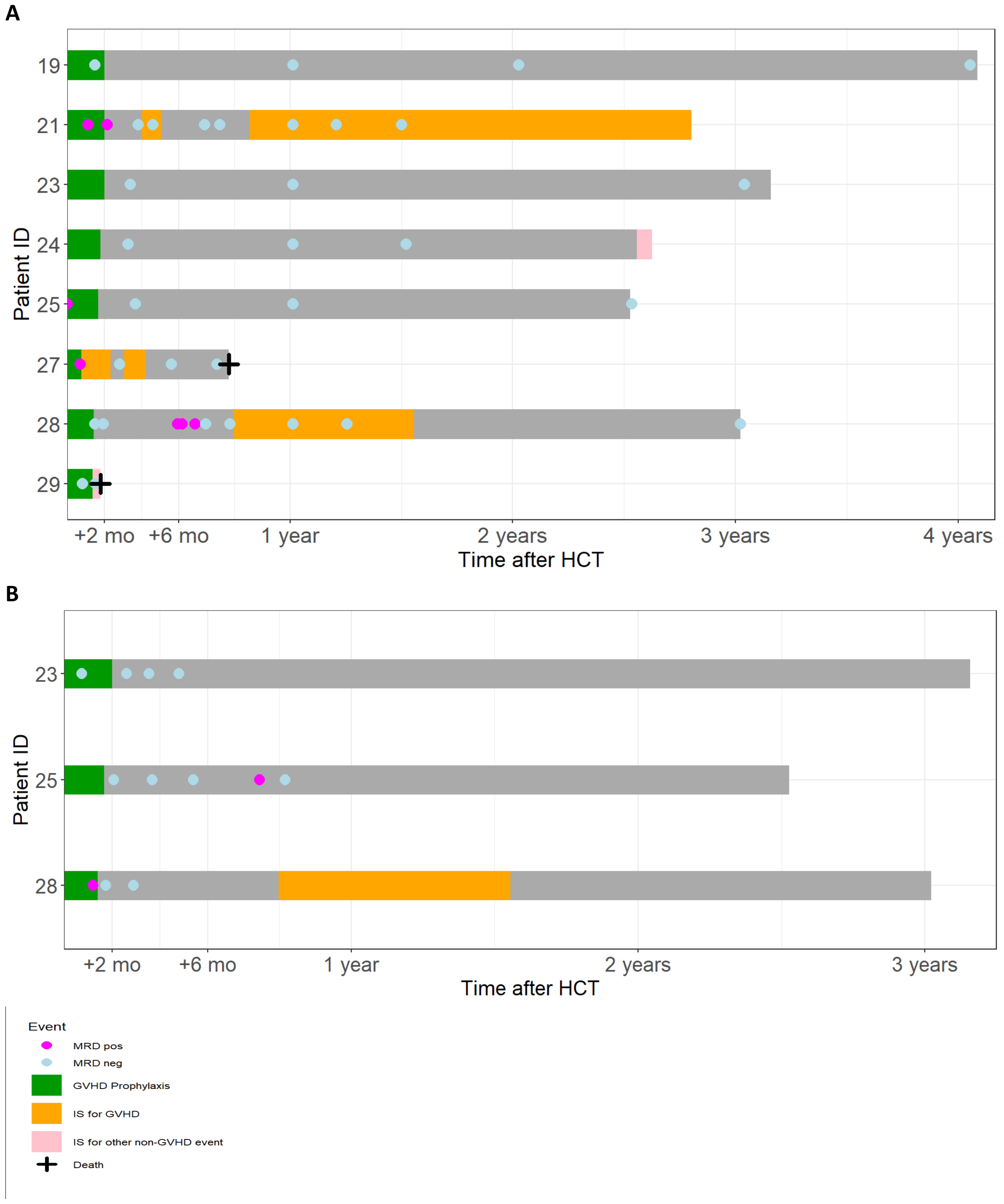

Supplemental Figure 1. Swimmer's plots of monitoring for sub-clinical disease, with survival duration shown in gray bars, periods of immunosuppression noted in bar shading, and instances of disease monitoring by flow cytometry in circles. Patient IDs match the patient IDs in Figure 1. A) Among patients with pre-HCT detection of disease in peripheral blood and marrow by flow cytometry and with post-HCT peripheral blood flow and/or marrow assessment (n=8), the detection or absence of disease by peripheral blood or marrow flow cytometry, serially over time, is shown. B) Among patients with post-HCT pleural effusions and with serial disease assessment by flow cytometry of pleural fluid (n=3), detection or absence of disease by flow cytometry of pleural fluid is shown. In all instances, this is low level detection of disease in the **absence** of other signs of disease. Patients with overt relapse/progression concurrent with detection of disease in peripheral blood, marrow, or pleural fluid are not shown.

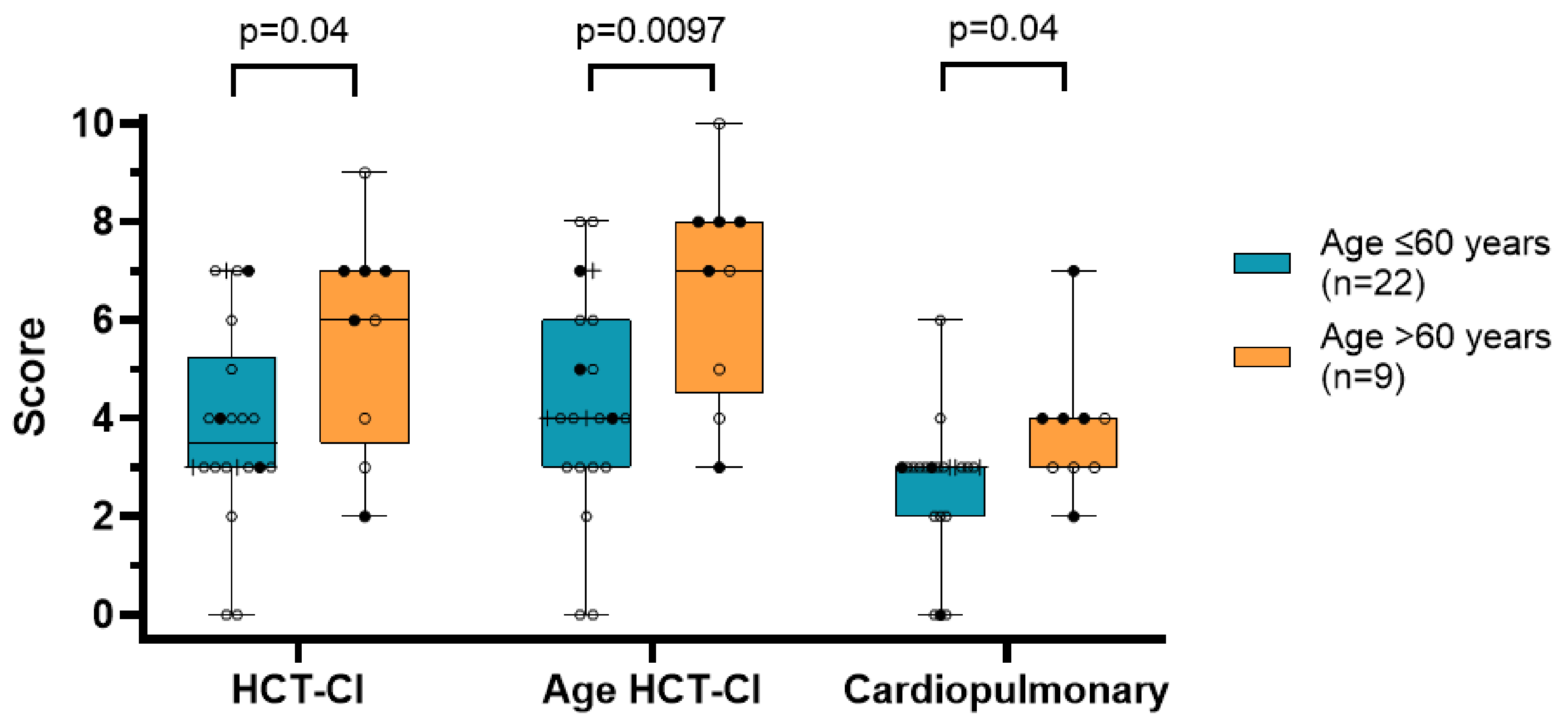

Supplemental Figure 2. Unpaired T-test comparison of pre-HCT factors by age, n=31; box and whisker plots denote minimum, first quartile, median, third quartile, and maximum scores. Age HCT-CI includes an additional point for age > 40 years. Cardiopulmonary HCT-CI score subset includes points attributed based on pulmonary function, arrhythmia, ejection fraction, and valvular disease. Transplant-related mortality is designated with a solid circle, and relapse/progression-related mortality is designated with a cross.

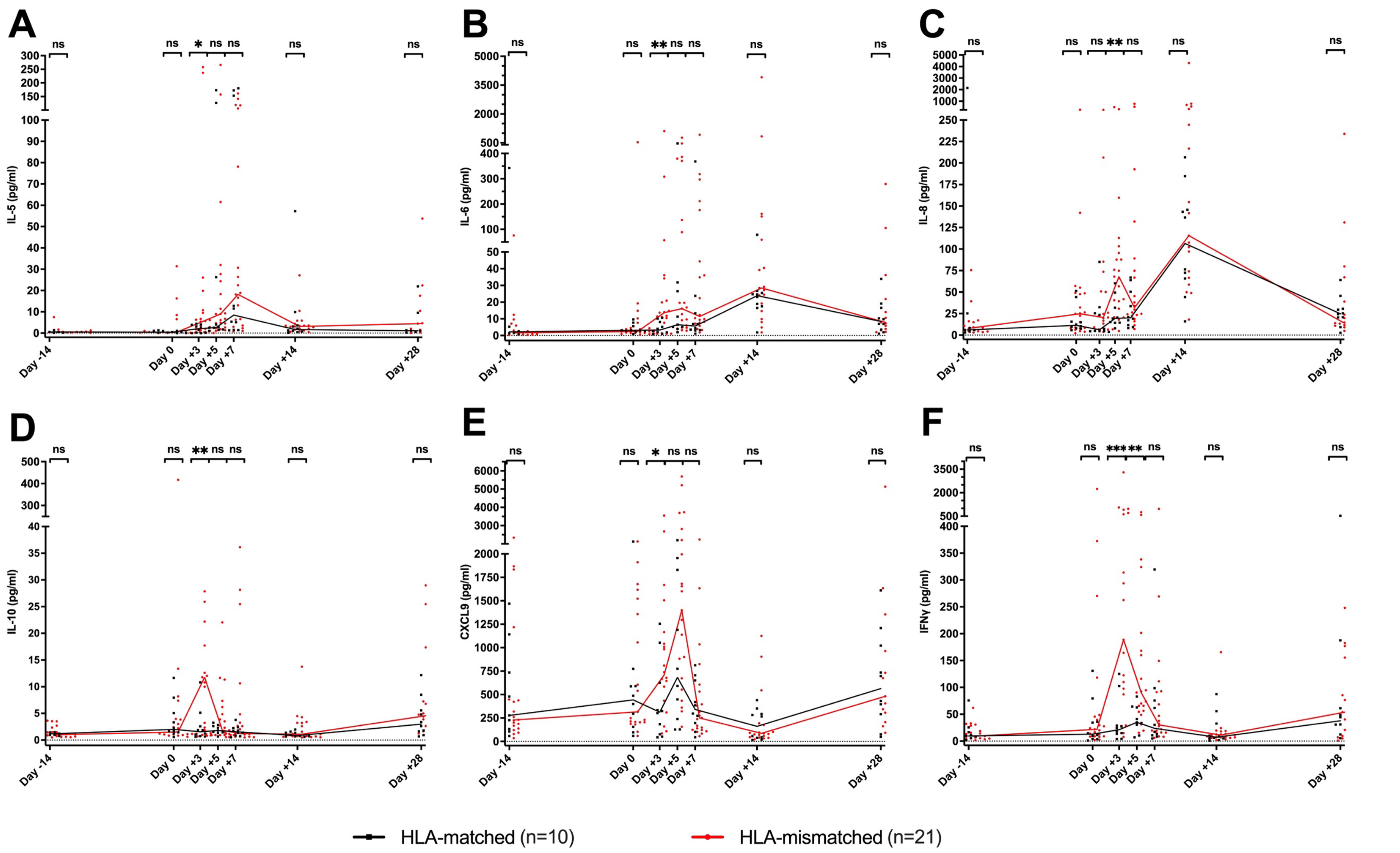

Supplemental Figure 3. Serial measurement of cytokine and chemokine levels in plasma, with comparison by Mann-Whitney test at each timepoint based on HLA-match. Levels of A) interleukin(IL)-5, B) IL-6, C) IL-8, D) IL-10, E) CXCL-9, F) interferon-γ at early HCT timepoints. Day 0 plasma specimens were drawn prior to cell administration. Post-transplantation cyclophosphamide (PTCy) is administered on day +3 and day +4 and plasma specimens were drawn on day +3 prior to PTCy. ns, not significant; *, $p<0.05$; **, $p<0.01$; ***, $p<0.001$.

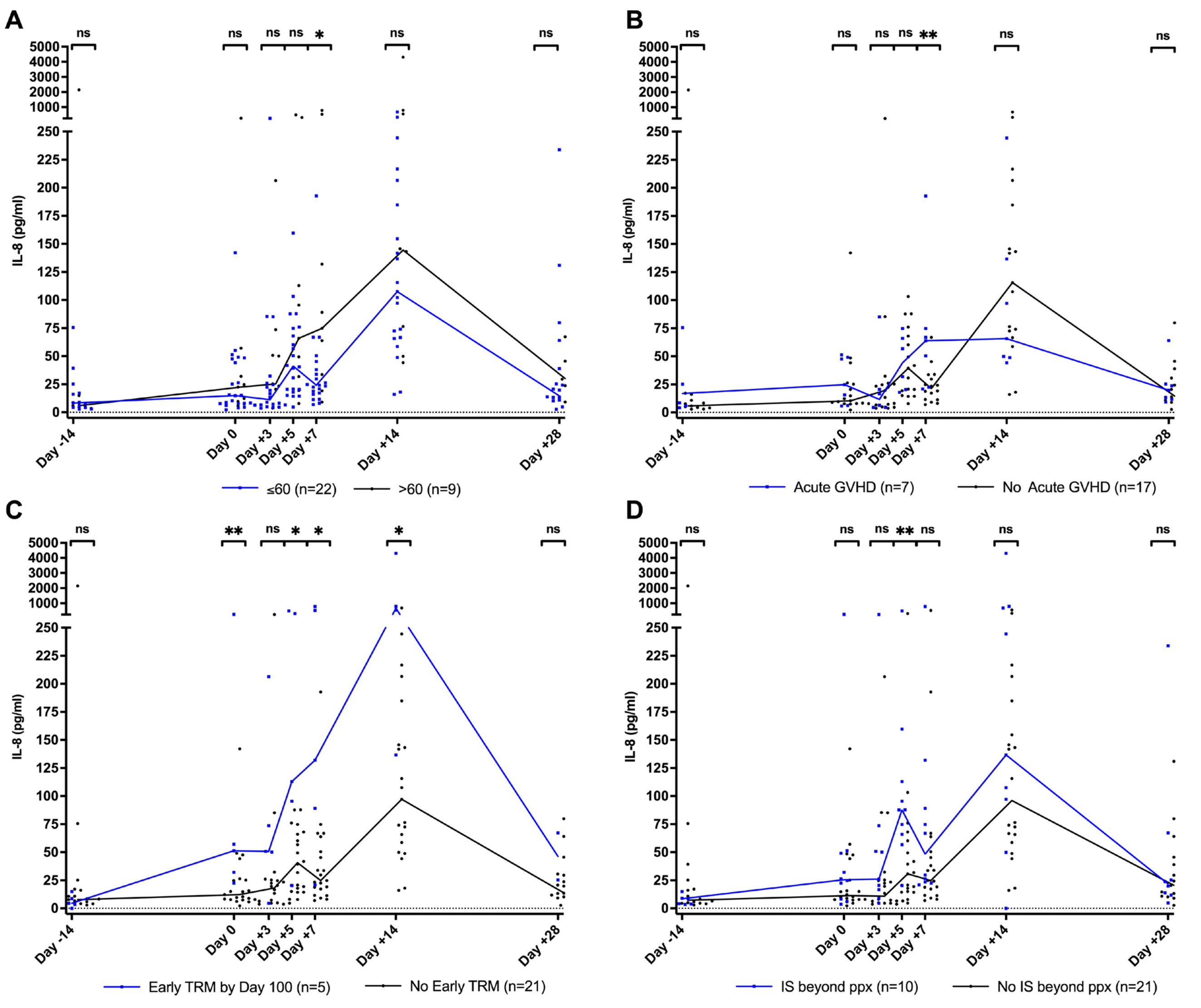

Supplemental Figure 4. Serial measurement of IL-8 levels in plasma, with comparison using Mann-Whitney tests at each timepoint. A) By age, B) By acute GVHD C), By early TRM (<100 days), D) By additional immunosuppression beyond prophylaxis by Day 100. Day 0 plasma specimens were drawn prior to cell administration. Post-transplantation cyclophosphamide (PTCy) is administered on day +3 and day +4 and plasma specimens were drawn on day +3 prior to PTCy. Patients without engraftment were excluded from GVHD analysis. Patients with relapse/progression were excluded from TRM analysis. ns, not significant; *, p<0.05; **, p<0.01.

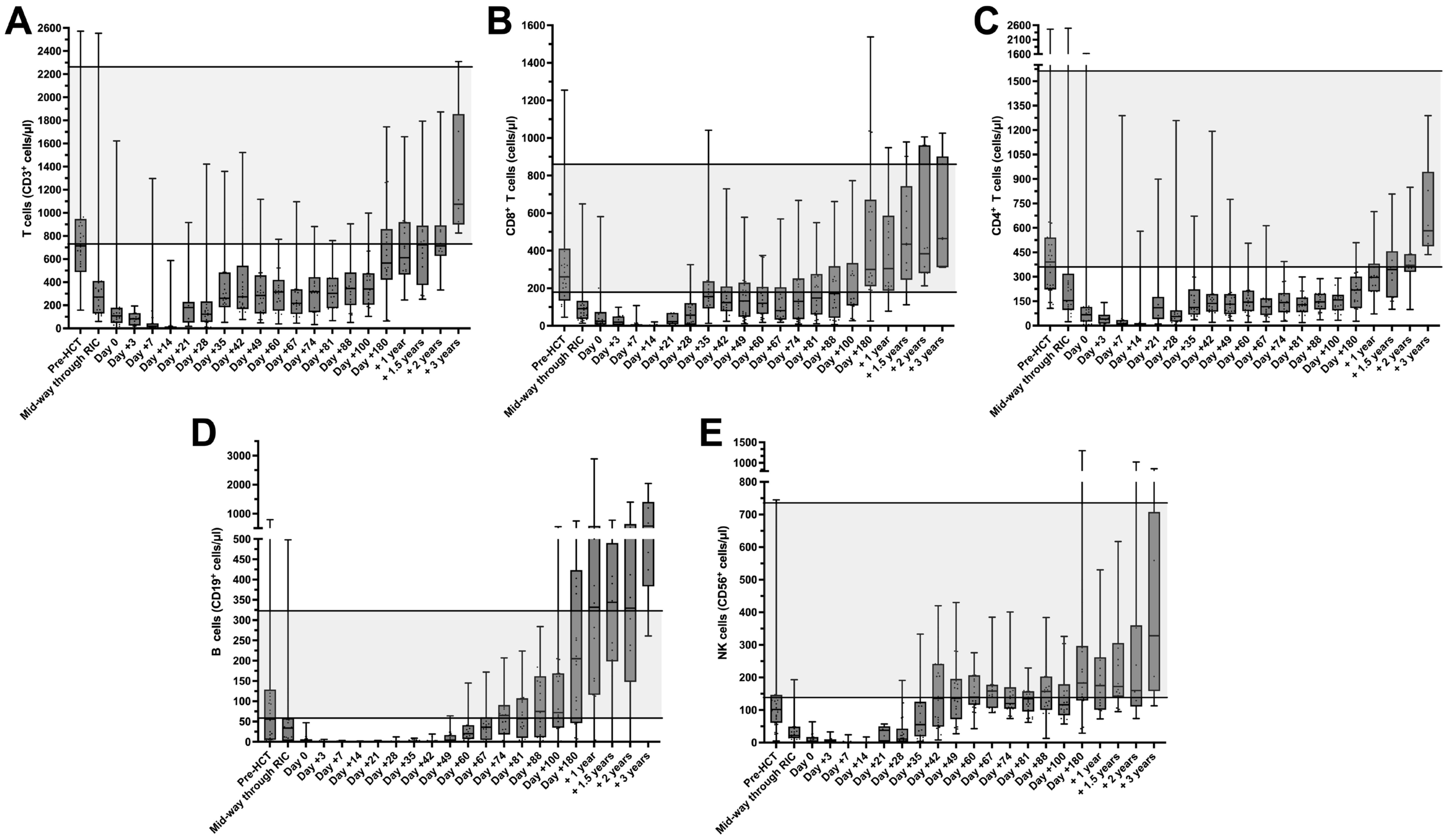

Supplemental Figure 5. Pre-HCT and post-HCT lymphocyte subset reconstitution for transplanted patients (n=31), with box-and-whisker plot denoting minimum, first quartile, median, third quartile, and maximum. Data points denote individual patient results. Upper and lower limit of institution-specific adult normal range are demarcated for each subset. Patients who experienced graft failure and/or death/treated relapse before day +180 are excluded from this analysis.

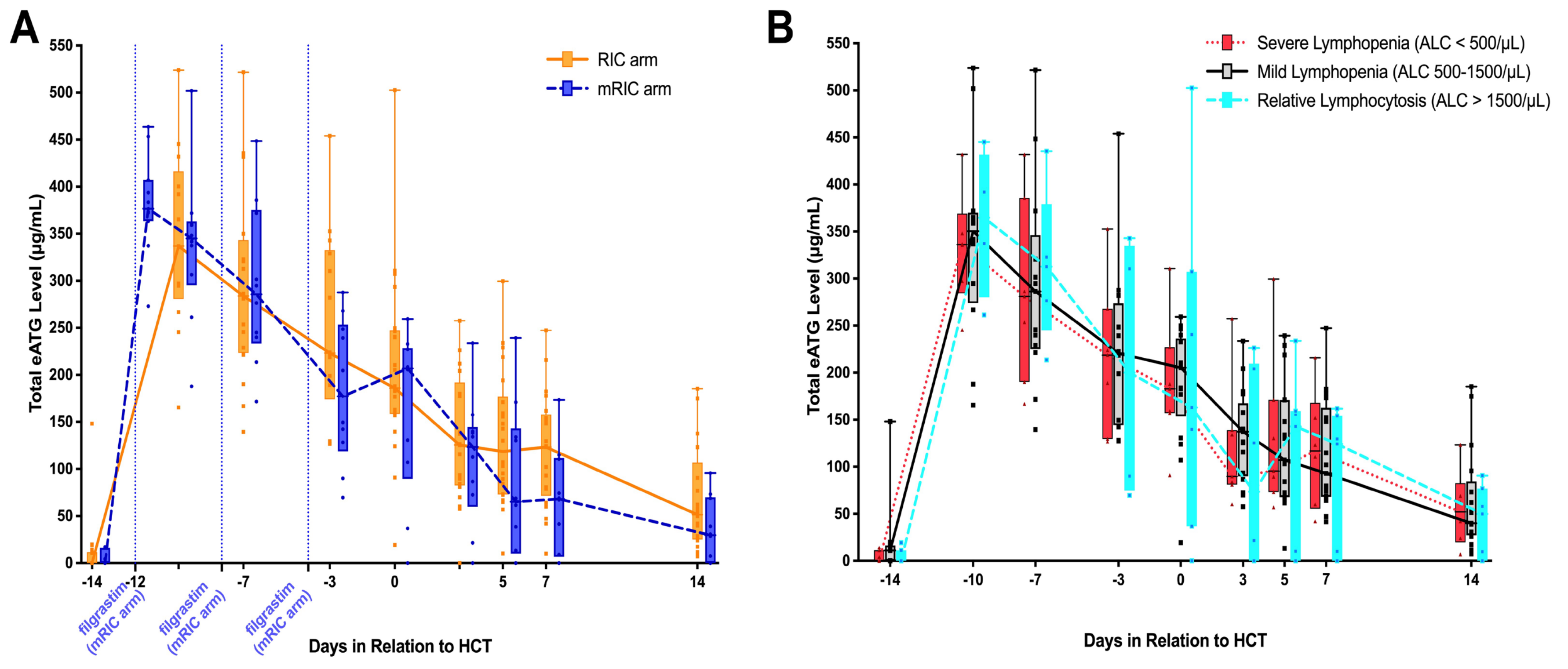

Supplemental Figure 6. Total plasma ATG levels by ELISA assay. Data points represent individual patients. Box and whisker plots denote minimum, first quartile, median, third quartile, and maximum. A) Total ATG levels compared between RIC (n=21) and mRIC (n=10) arms by Mann-Whitney test at each timepoint. mRIC patients received filgrastim on days -12, -8, -4, pre-HCT, whereas RIC arm patients did not receive filgrastim during conditioning. There was no significant difference in between arms in ATG level at any timepoint. B) Total ATG levels compared at each timepoint, grouped by pre-HCT lymphocyte count (severe lymphopenia, n=7; mild lymphopenia, n=17; relative lymphocytosis, n=7) and compared by Mann-Whitney test at each timepoint. No difference in AUC or levels at each timepoint.

Supplemental Figure 7. Patient plasma eATG binding, expressed as percent of parent, by cell subtype, graphed by study arm, with comparison between arms at each timepoint using Mann-Whitney tests. G-CSF was given to patients on the mRIC arm at days -12, -8, and -4, with the hypothesis that this would increase the clearance of e-ATG bound cells. Overall, the RIC arm had lower eATG binding of many cell subsets compared to the mRIC arm at many timepoints. A) CD4 T cells, B) CD4 Effector Memory T cells, C) CD4 Central Memory T cells, D) CD4 Stem Cell Memory T cells, E) CD8 T cells, F) CD8 Effector Memory T cells, G) CD8 Central Memory T cells, H) CD8 Stem Cell Memory T cells, I) Tregs. ns, not significant; *p<0.05; **p<0.01; ***p<0.001; ****p<0.0001.

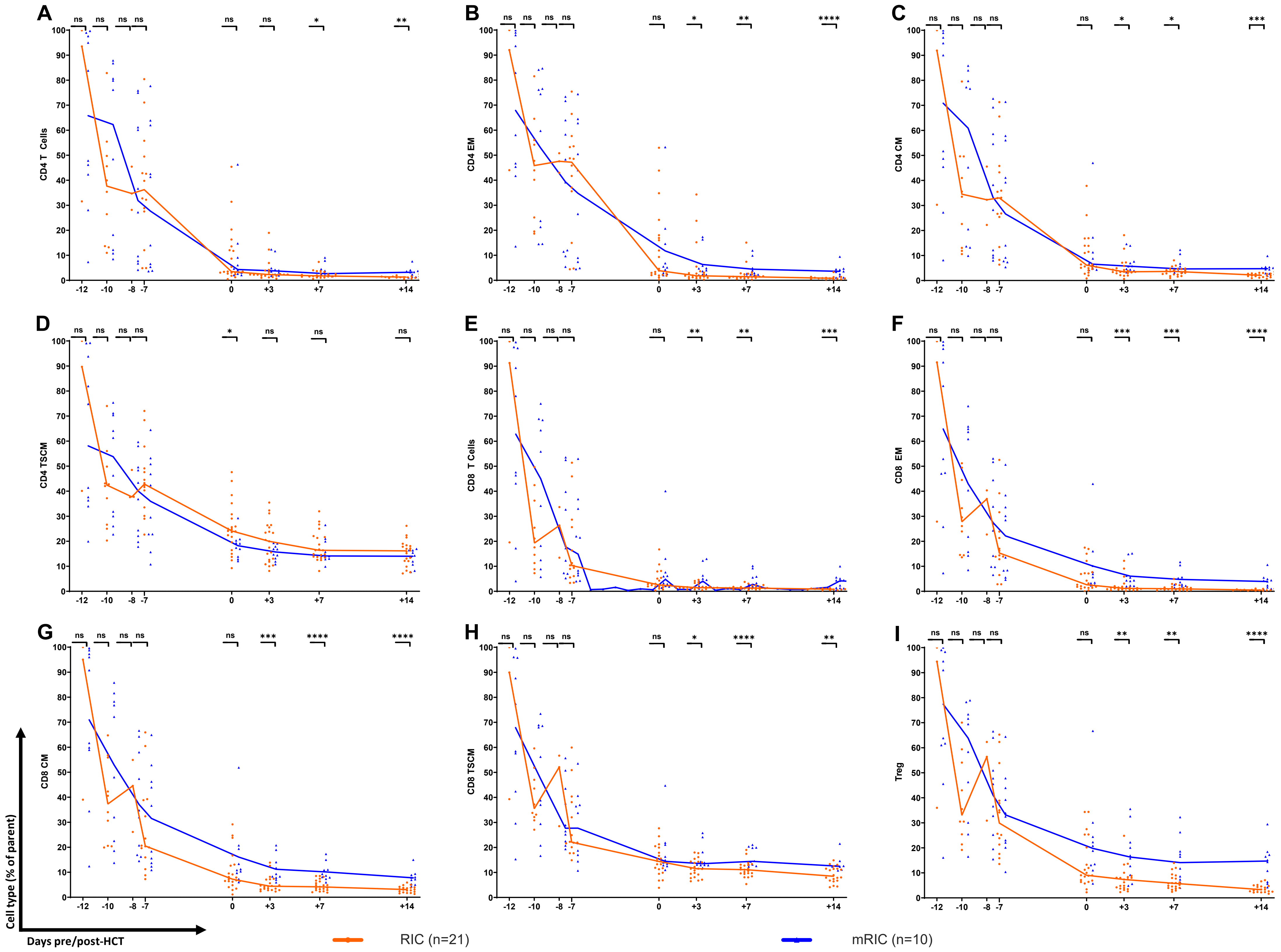

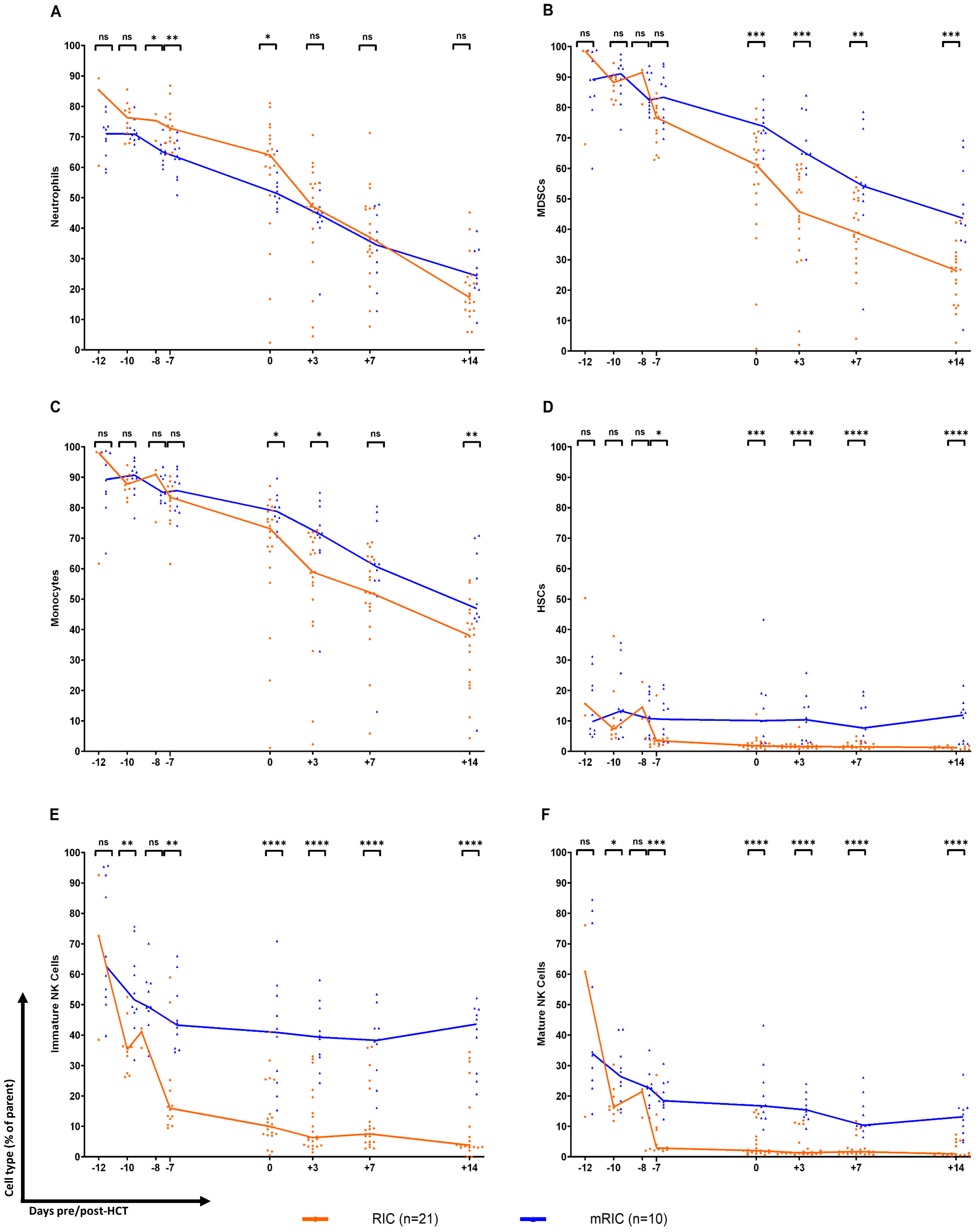

Supplemental Figure 8. Patient plasma eATG binding, expressed as percent of parent, by cell subtype, graphed by study arm, with comparison between arms at each timepoint using Mann-Whitney tests. Filgrastim was given to patients on the mRIC arm at days -12, -8, and -4, with the hypothesis that this would increase the clearance of e-ATG bound cells. Overall, the RIC arm had lower eATG binding of many cell subsets compared to the mRIC arm at many timepoints. A) Neutrophils, B) Myeloid Derived Suppressor Cells, C) Monocytes, D) Hematopoietic Stem Cells, E) Immature NK Cells, F) Mature NK Cells. ns, not significant; *p<0.05; **p<0.01; ***p<0.001; ****p<0.0001.

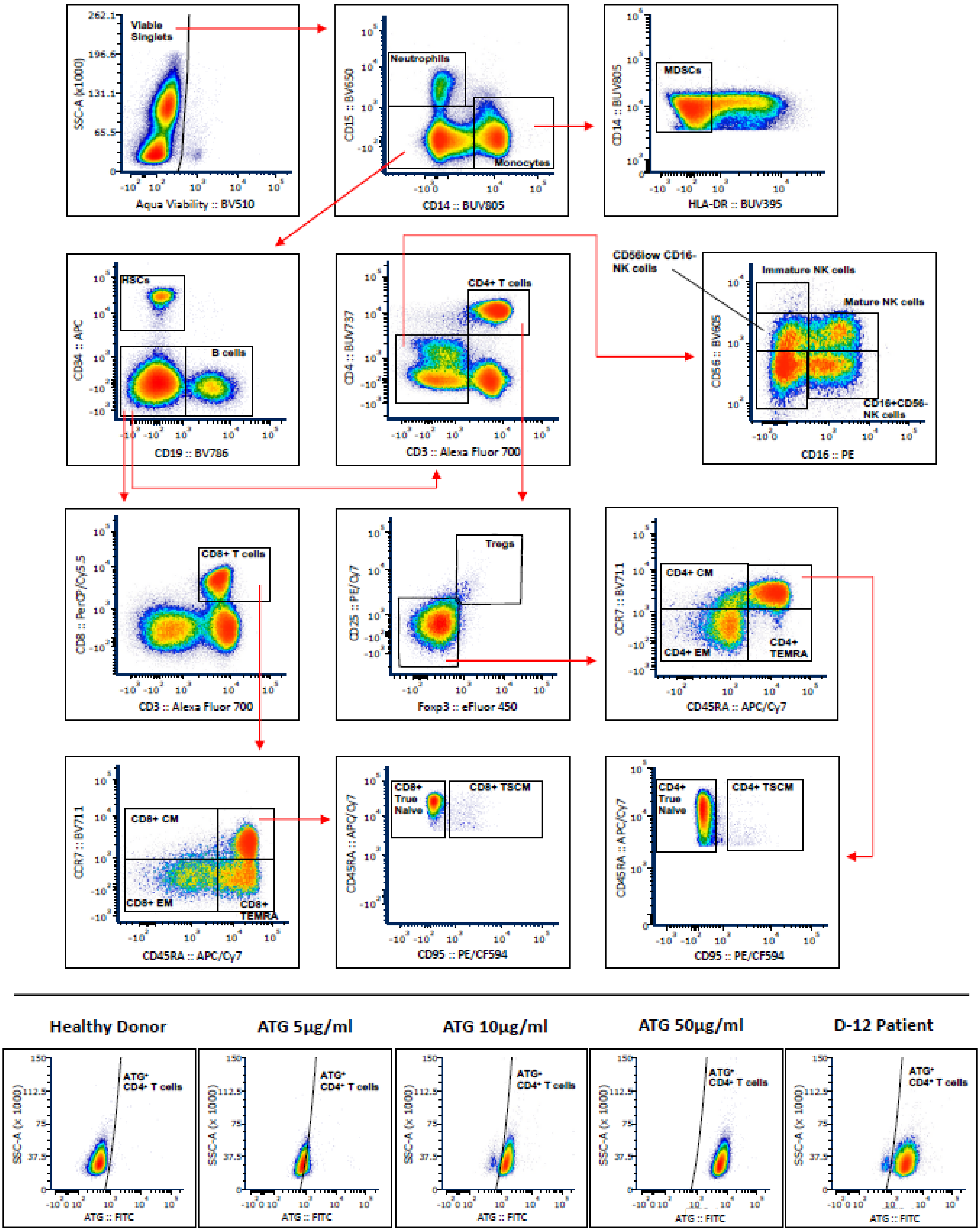

Supplemental Figure 9. Gating strategy for immune profiling via flow cytometry.

Section/topic	No	CONSORT 2025 checklist item description	Reported on page no.
**Title and abstract**			
Title and structured abstract	1a	Identification as a randomised trial	N/A (not randomized)
	1b	Structured summary of the trial design, methods, results, and conclusions	Pages 7-21
**Open science**			
Trial registration	2	Name of trial registry, identifying number (with URL) and date of registration	Page 18
Protocol and statistical analysis plan	3	Where the trial protocol and statistical analysis plan can be accessed	
Data sharing	4	Where and how the individual de-identified participant data (including data dictionary), statistical code and any other materials can be accessed	Page 21
Funding and conflicts of interest	5a	Sources of funding and other support (eg, supply of drugs), and role of funders in the design, conduct, analysis and reporting of the trial	Page 25
	5b	Financial and other conflicts of interest of the manuscript authors	Page 25
**Introduction**			
Background and rationale	6	Scientific background and rationale	Page 3-4
Objectives	7	Specific objectives related to benefits and harms	Page 19-20
**Methods**			
Patient and public involvement	8	Details of patient or public involvement in the design, conduct and reporting of the trial	n/a
Trial design	9	Description of trial design including type of trial (eg, parallel group, crossover), allocation ratio, and framework (eg, superiority, equivalence, non-inferiority, exploratory)	Page 18-19
Changes to trial protocol	10	Important changes to the trial after it commenced including any outcomes or analyses that were not prespecified, with reason	Page 18
Trial setting	11	Settings (eg, community, hospital) and locations (eg, countries, sites) where the trial was conducted	Page 18
Eligibility criteria	12a	Eligibility criteria for participants	Page 19
	12b	If applicable, eligibility criteria for sites and for individuals delivering the interventions (eg, surgeons, physiotherapists)	n/a
Intervention and comparator	13	Intervention and comparator with sufficient details to allow replication. If relevant, where additional materials describing the intervention and comparator (eg, intervention manual) can be accessed	Suppl Table 1
Outcomes	14	Prespecified primary and secondary outcomes, including the specific measurement variable (eg, systolic blood pressure), analysis metric (eg, change from baseline, final value, time to event), method of aggregation (eg, median, proportion), and time point for each outcome	Page 18-19
Harms	15	How harms were defined and assessed (eg, systematically, non-systematically)	Page 7-9
Sample size	16a	How sample size was determined, including all assumptions supporting the sample size calculation	Page 19-20
	16b	Explanation of any interim analyses and stopping guidelines	Page 19-20
Randomisation:			
Sequence generation	17a	Who generated the random allocation sequence and the method used	n/a
	17b	Type of randomisation and details of any restriction (eg, stratification, blocking and block size)	n/a

			Reported on page no.
Allocation concealment mechanism	18	Mechanism used to implement the random allocation sequence (eg, central computer/telephone; sequentially numbered, opaque, sealed containers), describing any steps to conceal the sequence until interventions were assigned	n/a
Implementation	19	Whether the personnel who enrolled and those who assigned participants to the interventions had access to the random allocation sequence	n/a
Blinding	20a	Who was blinded after assignment to interventions (eg, participants, care providers, outcome assessors, data analysts)	n/a
	20b	If blinded, how blinding was achieved and description of the similarity of interventions	n/a
Statistical methods	21a	Statistical methods used to compare groups for primary and secondary outcomes, including harms	Page 19-20
	21b	Definition of who is included in each analysis (eg, all randomised participants), and in which group	Page 19-20
	21c	How missing data were handled in the analysis	
	21d	Methods for any additional analyses (eg, subgroup and sensitivity analyses), distinguishing prespecified from post hoc	
**Results**			
Participant flow, including flow diagram	22a	For each group, the numbers of participants who were randomly assigned, received intended intervention, and were analysed for the primary outcome	n/a
	22b	For each group, losses and exclusions after randomisation, together with reasons	n/a
Recruitment	23a	Dates defining the periods of recruitment and follow-up for outcomes of benefits and harms	Page 18
	23b	If relevant, why the trial ended or was stopped	n/a
Intervention and comparator delivery	24a	Intervention and comparator as they were actually administered (eg, where appropriate, who delivered the intervention/comparator, how participants adhered, whether they were delivered as intended (fidelity))	n/a
	24b	Concomitant care received during the trial for each group	Suppl Table 1
Baseline data	25	A table showing baseline demographic and clinical characteristics for each group	Page 26 – Table 1
Numbers analysed, outcomes and estimation	26	For each primary and secondary outcome, by group:<br>● the number of participants included in the analysis<br>● the number of participants with available data at the outcome time point<br>● result for each group, and the estimated effect size and its precision (such as 95% confidence interval)<br>● for binary outcomes, presentation of both absolute and relative effect size	Page 19-20
Harms	27	All harms or unintended events in each group	Page 8-14
Ancillary analyses	28	Any other analyses performed, including subgroup and sensitivity analyses, distinguishing pre-specified from post hoc	Page 8-14
**Discussion**			**Page 15-18**
Interpretation	29	Interpretation consistent with results, balancing benefits and harms, and considering other relevant evidence	Page 17-18
Limitations	30	Trial limitations, addressing sources of potential bias, imprecision, generalisability, and, if relevant, multiplicity of analyses	

Citation: Hopewell S, Chan AW, Collins GS, Hróbjartsson A, Moher D, Schulz KF, et al. CONSORT 2025 Statement: updated guideline for reporting randomised trials. BMJ. 2025; 388:e081123. https://dx.doi.org/10.1136/bmj-2024-081123
© 2025 Hopewell et al. This is an Open Access article distributed under the terms of the Creative Commons Attribution License (https://creativecommons.org/licenses/by/4.0/), which permits unrestricted use, distribution, and reproduction in any medium, provided the original work is properly cited.

*We strongly recommend reading this statement in conjunction with the CONSORT 2025 Explanation and Elaboration and/or the CONSORT 2025 Expanded Checklist for important clarifications on all the items. We also recommend reading relevant CONSORT extensions. See www.consort-spirit.org.
