# Supplementary material for: Allogeneic hematopoietic cell transplantation in mature T- or NK-lymphomas: a phase II clinical trial
Source: Nat Commun. 2026 Apr 23;17:5606. doi: 10.1038/s41467-026-71461-5 (PMC13316028; doi:10.1038/s41467-026-71461-5)
Supplement: Supplementary file 2 — Reporting Summary [file 41467_2026_71461_MOESM2_ESM.pdf]

nature portfolio

Corresponding author(s): Dimana Dimitrova, MD

Last updated by author(s): 2025/12/10

Reporting Summary

Nature Portfolio wishes to improve the reproducibility of the work that we publish. This form provides structure for consistency and transparency in reporting. For further information on Nature Portfolio policies, see our Editorial Policies and the Editorial Policy Checklist.

Statistics

For all statistical analyses, confirm that the following items are present in the figure legend, table legend, main text, or Methods section.

n/a	Confirmed	
☐	☒	The exact sample size (*n*) for each experimental group/condition, given as a discrete number and unit of measurement
☐	☒	A statement on whether measurements were taken from distinct samples or whether the same sample was measured repeatedly
☐	☒	The statistical test(s) used AND whether they are one- or two-sided<br>*Only common tests should be described solely by name; describe more complex techniques in the Methods section.*
☐	☒	A description of all covariates tested
☒	☐	A description of any assumptions or corrections, such as tests of normality and adjustment for multiple comparisons
☐	☒	A full description of the statistical parameters including central tendency (e.g. means) or other basic estimates (e.g. regression coefficient) AND variation (e.g. standard deviation) or associated estimates of uncertainty (e.g. confidence intervals)
☐	☒	For null hypothesis testing, the test statistic (e.g. *F*, *t*, *r*) with confidence intervals, effect sizes, degrees of freedom and *P* value noted<br>*Give P values as exact values whenever suitable.*
☒	☐	For Bayesian analysis, information on the choice of priors and Markov chain Monte Carlo settings
☒	☐	For hierarchical and complex designs, identification of the appropriate level for tests and full reporting of outcomes
☒	☐	Estimates of effect sizes (e.g. Cohen's *d*, Pearson's *r*), indicating how they were calculated

Our web collection on statistics for biologists contains articles on many of the points above.

Software and code

Policy information about availability of computer code

Data collection	No software was used for data collection.
Data analysis	Prism 10 (10.3.1); R (4.3.2)

For manuscripts utilizing custom algorithms or software that are central to the research but not yet described in published literature, software must be made available to editors and reviewers. We strongly encourage code deposition in a community repository (e.g. GitHub). See the Nature Portfolio guidelines for submitting code & software for further information.

Data

Policy information about availability of data

All manuscripts must include a data availability statement. This statement should provide the following information, where applicable:

- Accession codes, unique identifiers, or web links for publicly available datasets
- A description of any restrictions on data availability
- For clinical datasets or third party data, please ensure that the statement adheres to our policy

The data that support the findings of this study are available as a Source Data file uploaded with

Research involving human participants, their data, or biological material

Policy information about studies with human participants or human data. See also policy information about sex, gender (identity/presentation), and sexual orientation and race, ethnicity and racism.

Reporting on sex and gender	Sex was reported in Table 1, nearly evenly distributed between male and female (48 vs 52%). Descriptive information was provided. Gender was not reported. No analysis was done based on sex due to the small sample size and no expected differences in clinical outcomes based on sex.
Reporting on race, ethnicity, or other socially relevant groupings	Race/ethnicity was self reported and included in Table 1 patient and graft characteristics. Descriptive information was provided with no subgroup analyses on clinical outcomes reported due to overall small sample size.
Population characteristics	Eligible patients were age >12 years with mature neoplasms of T- or NK-cell origin that were either relapsed/refractory, or of a risk category based on Prognostic Index for T-Cell Lymphoma score or clinical practice guidelines wherein upfront HCT in first-remission is considered an appropriate therapeutic option. Overall, patient age ranged from 13 to 71 years, with diagnoses including angioimmunoblastic T cell lymphoma, Peripheral T cell lymphoma not otherwise specified, Alk-negative anaplastic large cell lymphoma, EBV+ NK/T cell lymphoma, cutaneous T cell lymphoma, adult T cell lymphoma, hepatosplenic gamma delta T cell lymphoma, Monomorphic epitheliotropic intestinal T cell lymphoma. Patients had progressive disease, complete or partial remission after primary refractory, second or beyond remission after relapse.
Recruitment	Patient were recruited through referral from their primary oncologist/hematologist or via self referral. No self-selection bias that we are aware of.
Ethics oversight	National Institutes of Health Institutional Review Board (IRB)

Note that full information on the approval of the study protocol must also be provided in the manuscript.

Field-specific reporting

Please select the one below that is the best fit for your research. If you are not sure, read the appropriate sections before making your selection.

☒ Life sciences ☐ Behavioural & social sciences ☐ Ecological, evolutionary & environmental sciences

For a reference copy of the document with all sections, see nature.com/documents/nr-reporting-summary-flat.pdf

Life sciences study design

All studies must disclose on these points even when the disclosure is negative.

Sample size	Sample size was 31. Patients were transplanted on the RIC arm, followed by a modified RIC (mRIC) arm upon full accrual to the RIC arm. The study was statistically designed as a Simon 2-Stage to have 80% power in the RIC arm to determine whether there is a difference between a 45% 1-year PFS probability and an improved 70% 1-year PFS probability, with a one sided 0.10 alpha level test, using the method of Brookmeyer and Crowley31; the Simon 2-Stage design was not repeated for the subsequent mRIC arm given progression to Stage 2 in the RIC arm. Participants enrolled in the mRIC arm were included up to enrollment pause August 23, 2022 for data analysis. Sample size for exploratory endpoints varied because they were inherently tied to patient outcomes (i.e., samples were not available if the patient died).
Data exclusions	Data were locked for analysis on July 18, 2024. Survival endpoint probabilities were estimated using the Kaplan-Meier method with 95% confidence intervals (CIs). CuI of TRM, GVHD, CMV infection, and GF were estimated by competing-risk analysis using Gray's method36. Death was a competing risk for all analyses except TRM; PTCL relapse/progression was a competing risk for TRM. As many patients had active disease pre-HCT and disease status was not fully assessed at times of critical illness/decompensation in the early post-HCT period, the data coding approach was to consider these deaths as TRM even though some of these patients may have also had active/progressive disease at the time of death. GF was considered a competing risk for CMV infection, aGVHD, and cGVHD. Chronic GVHD served as an additional competing risk for aGVHD.
Replication	Statistical analyses were replicated at least 3 times with consistent findings/results. Primary source data from medical charts also verified at least 3 times.
Randomization	Phase 2 study. No randomization was performed.
Blinding	No randomization or blinding performed on analysis of primary and secondary endpoints. For exploratory analyses, lab performing cytokine assays was blinded. Other analyses were not blinded as they were performed in batches according to timing of accrual, with mRIC arm participants following RIC arm participants.

Reporting for specific materials, systems and methods

We require information from authors about some types of materials, experimental systems and methods used in many studies. Here, indicate whether each material, system or method listed is relevant to your study. If you are not sure if a list item applies to your research, read the appropriate section before selecting a response.

Materials & experimental systems

n/a	Involved in the study	
	x	Antibodies
x		Eukaryotic cell lines
x		Palaeontology and archaeology
x		Animals and other organisms
	x	Clinical data
x		Dual use research of concern
x		Plants

Methods

n/a	Involved in the study	
x		ChIP-seq
	x	Flow cytometry
x		MRI-based neuroimaging

Antibodies

Antibodies used

Rabbit anti-horse IgG (Fab'2) (LS-C60476, 2mg/ml. 1:400 for coating. Lot#177112. LifeSpan BioSciences, Seattle, WA).
Series diluted hATG (Atgam, Upjohn, Kalamazoo, MI)
Biotin-conjugated goat anti-horse IgG (Fab'2) antibody at 1:100,000
Polyclonal goat anti-horse IgG (Fab'2) secondary antibody (Lifespan Biosciences; cat. #LS-C60461-2).
BUV395 mouse anti-human HLA-DR (clone G46-6; BD Biosciences; cat #564040),
BUV737 mouse anti-human CD4 (clone SK3; BD Biosciences; cat #612748),
BUV805 mouse anti-human CD14 (clone M5E2; BD Biosciences; cat #612902),
BV605 mouse anti-human CD56 (clone 5.1H11; Biolegend; cat #362538),
BV650 mouse anti-human CD15 (clone HI98; BD Biosciences; cat #564232),
BV711 mouse anti-human CD197 (clone G043H7; Biolegend; cat #353228),
BV786 mouse anti-human CD19 (clone SJ25C1; BD Biosciences; cat #563325),
PerCP/Cy5.5 mouse anti-human CD8 (clone SK1; Biolegend; cat #344710),
PE mouse anti-human CD16 (clone 3G8; Biolegend; cat #302008),
PE-CF594 mouse anti-human CD95 (clone DX2; BD Biosciences; cat #562395),
PE/Cy7 mouse anti-human CD25 (clone BC96; Invitrogen; cat #25-0259-42),
APC mouse anti-human CD34 (clone 581; BD Biosciences; cat #555824),
AF700 mouse anti-human CD3 (clone UCHT1; Biolegend; cat #300424),
APC/Cy7 mouse anti-human CD45RA (clone HI100; Biolegend; cat #304128),
eFluor 450 rat anti-human Foxp3 (clone PCH101; Invitrogen; cat #48-4776-42).
FITC mouse anti-human CD45 (Biolegend; cat #304006),
PE mouse anti-human CD56 (BD; cat #555516),
PerCP/Cy5.5 mouse anti-human CD16 (Biolegend; cat #302028),
PECy7 mouse anti-human CD14 (Ebioscience; cat #25-0149-42),
APC mouse anti-human CD19 (Biolegend; cat #302212),
Alexa Fluor700 mouse anti-human CD3 (BD; cat #557943),
BV410 mouse anti-human CD4 (Biolegend; cat #300518),
eFluor 450 mouse anti-human CD8 (Ebiosciences; cat #48-0088-42).
FITC mouse anti-human CD45 (Biolegend; cat #304006),
PE mouse anti-human CD56 (Miltenyi Biotec; cat #130-113-312),
PECy7 mouse anti-human CD14 (eBioscience; cat #25-0149-42),
APC mouse anti-human CD19 (Biolegend; cat #302212),
Alexa Fluor700 mouse anti-human CD3 (BD; cat #557943),
eFluor 450 mouse anti-human CD8 (eBioscience; cat #48-0088-42),
BV410 mouse anti-human CD4 (Biolegend; cat #317444).

Validation

Validation performed by manufacturer.

Clinical data

Policy information about clinical studies

All manuscripts should comply with the ICMJE guidelines for publication of clinical research and a completed CONSORT checklist must be included with all submissions.

Clinical trial registration

NCT03922724

Study protocol

Protocol provided with initial submission. Can be requested at anytime from corresponding author.

Data collection

April 18, 2019 to July 18, 2024

Outcomes

The primary endpoint was progression-free survival (PFS) at 1-year post-HCT.
Other endpoints were analyzed across all patients, or in exploratory subgroups. Secondary endpoints included overall survival (OS) and the cumulative incidences (CuI) of acute GVHD (aGVHD), chronic GVHD (cGVHD), graft failure (GF), progression/relapse, infectious complications, and TRM. Acute and cGVHD were diagnosed and graded using standard criteria32,33. Neutrophil recovery was defined as the first of three days when a post-nadir absolute neutrophil count (ANC) reached ≥500/mm3. Engraftment syndrome

was defined using Spitzer criteria. Primary graft failure was defined as <5% donor myeloid chimerism in blood and/or bone marrow on all evaluations up to and including day +60, in the absence of a recurrent marrow malignancy. Secondary graft failure was defined as having achieved neutrophil recovery and initial blood or marrow donor myeloid chimerism ≥5%, declining to <5% on subsequent measurements. The disease status of the patient's PTCL pre- and post- HCT was evaluated with testing and imaging as appropriate for the PTCL subtype and manifestations.

Plants

Seed stocks

Report on the source of all seed stocks or other plant material used. If applicable, state the seed stock centre and catalogue number. If plant specimens were collected from the field, describe the collection location, date and sampling procedures.

Novel plant genotypes

Describe the methods by which all novel plant genotypes were produced. This includes those generated by transgenic approaches, gene editing, chemical/radiation-based mutagenesis and hybridization. For transgenic lines, describe the transformation method, the number of independent lines analyzed and the generation upon which experiments were performed. For gene-edited lines, describe the editor used, the endogenous sequence targeted for editing, the targeting guide RNA sequence (if applicable) and how the editor was applied.

Authentication

Describe any authentication procedures for each seed stock used or novel genotype generated. Describe any experiments used to assess the effect of a mutation and, where applicable, how potential secondary effects (e.g. second site T-DNA insertions, mosiacism, off-target gene editing) were examined.

Flow Cytometry

Plots

Confirm that:

- [x] The axis labels state the marker and fluorochrome used (e.g. CD4-FITC).
- [x] The axis scales are clearly visible. Include numbers along axes only for bottom left plot of group (a 'group' is an analysis of identical markers).
- [x] All plots are contour plots with outliers or pseudocolor plots.
- [x] A numerical value for number of cells or percentage (with statistics) is provided.

Methodology

Sample preparation

Peripheral blood stem cells (PBSCs) were ficolled, aliquoted, and cryopreserved. One aliquot from the same PBSC product (single donor) was thawed on each day of sample processing.

Instrument

The model is BD LSRFortessa Cell Analyzer.

Software

FCS Express

Cell population abundance

Viable singlet peripheral blood stem cells (PBSCs) were gated according to the gating strategy provided, where the number of cells ranged from 100 to 1,000 in low abundance immune cell populations (Tregs; Immature NK cells; CD4+ TSCMs, CM, and TEMRA cells; CD8+ TSCMs and CM cells), from 1,000 to 10,000 in medium abundance immune cell populations (HSCs; CD4+ T cells; CD4+ EM and true naïve cells; CD8+ TEMRA, EM, and true naïve cells; mature NK cells) and from 10,000 to 70,000 in high abundance immune cell populations (B cells, CD8+ T cells, MDSCs, Neutrophils, Monocytes). No flow cytometric sorting was performed.

Gating strategy

Cells were gated through SSC-A/FSC-A, SSC-H/SSC-W, and FSC-H/FSC-W to obtain singlets. Afterwards, singlets were subgated via the strategy provided in the attached figure to define the immune cell subsets of interest.

- [x] Tick this box to confirm that a figure exemplifying the gating strategy is provided in the Supplementary Information.
