# Supplementary material for: Allogeneic hematopoietic cell transplantation in mature T- or NK-lymphomas: a phase II clinical trial
Source: Nat Commun. 2026 Apr 23;17:5606. doi: 10.1038/s41467-026-71461-5 (PMC13316028; doi:10.1038/s41467-026-71461-5)
Supplement: Supplementary file 3 — Transparent Peer Review File [file 41467_2026_71461_MOESM3_ESM.pdf]

nature portfolio

Peer Review File

Allogeneic Hematopoietic Cell Transplantation in Mature T- or NK-lymphomas: A Phase II Clinical Trial

Corresponding Author: Dr Dimana Dimitrova

This file contains all reviewer reports in order by version, followed by all author rebuttals in order by version.

Version 0:

Reviewer comments:

Reviewer #1

(Remarks to the Author)
The authors present results from a prospective trial of allo-transplant for patients with a wide variety of T and NK cell lymphomas. They utilize a platform that incorporates distal horse ATG and PTCy as part of the treatment schema. Results demonstrate PFS of over 50% in what appears to be a relatively high risk group of patients which is at least as good as that presented in prior reports. The regimen was rather toxic however with 15 of 31 patients requiring intubation and 5 requiring renal replacement therapy. It does appear that patients with what was termed refractory disease could achieve prolonged remissions with this approach.

1) Toxicity and non relapse mortality were relatively high. Do the authors attribute this to the regimen or clinical status of the recipients?

2) What was the purpose of the ATG in this trial? Was it to promote engraftment, prevent, GVH, or deplete tumor cells pre-transplant. How do the authors assess the impact, positive or negative of the ATG as levels were low at Day 0.

3) The authors report that compared to younger patients, patients >60 years had higher day +7 levels of IL-8 ($p=0.03$) and CXCL9 ($p=0.05$), with a tendency toward elevation in day +7 IFN-γ ($p=0.07$). What impact do the authors think that has on the observed clinical findings?

4) In the Discussion, the authors state a recommendation that "investigators from Johns Hopkins advocate PBSC is the preferred graft source in PTCL and we echo that recommendation." Do the authors have evidence from their own data supporting this?

5) As the most striking results from this report are those in the 19 patients with refractory disease, please provide more data about these patients.

6) The manuscript, particularly the discussion could be shortened significantly.

Reviewer #2

(Remarks to the Author)
Comments to the authors:
Rechache et al describe a prospective clinical trial in patients with PTCL, including patients with progressive disease at the time of hematopoietic cell transplant (HCT), targeting a patient population in whom there is a paucity of prospective HCT-based studies. The approach taken is aimed to address the challenge of progressive disease prior to HCT, and while it does show some promise it is also associated with significant incidence of early transplant-related mortality (TRM). This makes it difficult to immediately translate the findings into practice, and would require further study to best ascertain which patients would be most suitable for this sort of transplant. In our opinion, the overall effort is commendable and the methodology is sound. The manuscript can be acceptable for publication after major revisions, and would be suitable for a journal for HCT providers treating PTCL.
Specific comments are below:
Major Comments

1. It is commendable for the authors to provide an approach for patients who otherwise would not have access to curative therapy. Unfortunately there was a high incidence of early TRM, particularly for a regimen that is considered to be one of reduced intensity (RIC). The authors should put into context how this incidence of early TRM compares to most RIC regimens based on retrospective registry data.
The high early TRM makes it challenging to integrate the approach described into standard practice. The authors should present additional PFS and OS curves, dichotomizing by disease type (for example, combination of AITL, PTCL-NOS, Alk-neg ALCL vs the rest; PTCL with progressive disease at the time of HCT vs the rest), age (< 60 vs >= 60) and HCT-CI score (0-3 vs > 3) in order to provide more insight as to what baseline characteristics may have been associated with outcomes. The authors note there was a difference in TRM between patients age < 60 vs >= 60, but it is not clear why this would be the case for a RIC regimen – did the older patients have more aggressive disease, more prior therapies, more baseline comorbidities? Were there any specific subcomponents of the HCT-CI (for example baseline cardiovascular comorbidities, pulmonary comorbidities, etc) that were associated with TRM? Is there a patient subgroup that would have the greatest benefit relative to risk from the approach described in this manuscript?
2. We note that an important limitation of the data is that due to the high incidence of early TRM, it is difficult to make any statements about the graft-vs-tumor effect. Since an evaluation for disease relapse could not be done at the time of clinical decompensation, and since the latter was often early, could it have been the case that many patients with active disease at the time of HCT would have eventually relapsed had they not died for another reason? The authors should soften their statements about the graft-vs-tumor effect throughout the manuscript given it could not be evaluated in a sizeable portion of the treated patients.
3. As we understand it, the hypothesis with the use of hATG was to provide some measure of disease burden reduction alongside the other conditioning regimens, and with aim to have cytotoxic levels clear out before day 0. But it is not clear what is a ‘therapeutic level’ and what is a ‘cytotoxic level’ of hATG based on the presented data. The authors should indicate these levels in Figure 5 and provide additional references for these levels. Furthermore, many HCT protocols use ATG, and many give it more proximally to day 0. Please explain how the data on eATG levels with the more distal administration compares to other protocols where the ATG is given more proximally.
4. A recent publication (Admiraal R et al, Blood Advances 2025) indicated that model-based dosing of ATG can lead to improved survival and reduced morbidity, with the model based in part on the ALC. The authors show the eATG levels relative to ALC pre-HCT in Figure 5B, but there does not appear to be any difference based on the baseline ALC – please discuss this in the context of the recent Blood Advances paper.
5. It is notable as well that the cumulative incidence of severe chronic GVHD of 23% is high for PTCY-based regimens with RIC conditioning. One possibility is that the early immune suppression discontinuation, while aiming to promote a graft-vs-tumor effect, came at a significant cost. The authors should describe the organ manifestations in the patients with chronic GVHD. Among surviving patients beyond 1 year, what was the incidence of severe chronic GVHD? In addition, according to the text there were 11 patients in whom sirolimus was stopped even earlier than the planned day +60. The authors should provide an explanation as to why this was, and conduct a subgroup analysis comparing outcomes of these patients relative to others with respect to PFS, OS, TRM, and incidence and severity of chronic GVHD.
6. The 60-day cumulative incidence of graft failure of 10% is important to discuss. Please discuss this in the context of graft failure rates in HLA mismatched transplants in general.
7. In supplemental Table 2, there appears to be a difference (though not clear if statistically significant based on small numbers) between outcomes of HLA matched and HLA mismatched transplants. What is the hypothesis for this?
8. It was hypothesized that filgastrim in the context of the mRIC regimen may augment the clearance of hATG-bound cells, but this did not appear to be the case as per the data in Figure 5A. So then why in the comparison of PFS between the two arms does there appear to be a visual difference between RIC and mRIC(Figure 2A), with a seemingly rapid drop in the PFS curve in the mRIC arm? The authors should comment on this.
9. To add to the point above, what is the hypothesis about the differences in reconstitution of T and NK cell populations between RIC and mRIC given that up-front filgastrim did not augment the clearance of hATG-bound cells?
Minor Comments
1. Please put the number of patients evaluated at each time point in each figure panel as it helps to consider how many were at risk for a particular event. This was done in some plots and not others. Please do this for all survival curves.
2. The fonts have to be consistent across all plots. For example, in Figure 3, the font sizes appear different in each subplot. We can barely read some of the numbers. Figure 3D should go in the supplemental as it is only showing data for 1 patient, and this can help with the font sizes in the other plots. Please re-make the figures and use a consistent font size across all plots. This may mean putting some more of them in the supplemental.
3. Figure 4 could go into the supplemental, and again, please remake the plots so that the fonts are not so tiny. We cannot tell if there was any statistical analysis done in these figures to compare the RIC and mRIC arm.
4. We suggest for the swimmer’s plot (Figure 6) to be moved up earlier, possibly as Figure 2.
5. Supp Figure 2: this figure needs to be revised. The font is way too small. Put “n=” as an indication of the number of patients evaluated under each time point.
6. Supp Figure 3: the font is way too small. We think there were some statistical comparisons done, but we can’t tell what is significant or not.

Reviewer #3

(Remarks to the Author)
I co-reviewed this manuscript with one of the reviewers who provided the listed reports. This is part of the Nature Communications initiative to facilitate training in peer review and to provide appropriate recognition for Early Career Researchers who co-review manuscripts.

Reviewer #4

(Remarks to the Author)
Summary:
This manuscript decribes a single arm, single institution, prospective experience using allo-HCT to treat T and NK cell lymphomas. The majority of these patients had high risk disease, many had persistent disease even at high levels. This was an overall high risk cohort in respect to comorbid conditions and need for mismatched donors. The first group of patient had a RIC allo as described in the paper, and the second cohort with a modified RIC platform with GCSF to modulate effect of ATG.

Correlative studies including immune reconstitution by flow cytometry and serum cytokines were done.

Results were promising, underscoring the graft versus tumor effect in this disease, and the fact that good outcomes can be attained even in patients with active disease. The TRM was concerningly high in the older patients, which likely renders this platform best used in younger patients.

Correlatives were well done and communicated. However, I would say there were relatively few clear signals from the analysis, which is common for transplant studies. This reflects how challenging it is to do and analyze these data and also the differences in the biology of patients before and peri-transplant. But it is important to continue to do these studies as we get more insight into post transplant biology in the context of relapse, GVHD, and long remissions.

This manuscript has several strengths. It is remarkably well written and concise. Relevant literature is reviewed. This platform is very promising in a disease that can be difficult to treat and effect young people. The patient population was high risk, more underscoring the good outcomes.

I believe that this paper is appropriate for publication with few edits.

Major:
1. Figure 2 – I think this composite figure is misleading. Looking at Penal !, I expected the rest of the figures to be red RIC and blue mRIC. I think it is most important to differentate panel A from the others, since in reading the manuscript you expect comparisons to be RIC vs mRIC, which is not the case for any other panels. Each other panel makes a different comparison. I suggest changing he colors for panels B C and D. I also suggest having the legend in the exact same place in all four panels and making it bigger.

I understand why the composite figure is being used, but I would be careful to make it abundantly clear that all these comparisons are not RIC vs mRIC.

2. Figure 3 – the colors don't work. In most places in panels B and C there is no color applied to the bars, since the bars and nonexistant.

3. For the CXCL9 and IL8 elevations in older patients, any correlation to the ones who had poor outcomes?

4. Please add a sentence on why ATL patients were excluded on the mRIC arm.

5. Was tocilizumab used appropriately? For example, in patients with grade 2 CRS or perhaps persistent fevers? We often find it is used per MD discretion and at times in patients with grade 1 CRS but not in patients with grade 2 CRS.

a. Why were toci patients excluded from serum IL-6 measurements? Does not need to be in the manuscript necessarily but curious what your cytokine panels revealed. Were IL-6 levels higher?

6. In the Supplement, sample size for RIC arm was 21 (enrolled) and sample sie for mRIC arm was 20. Why did you publish early? Is the trial still enrolling? Please clarify in the manuscript.

Minor:
1. Grammatical issues:
a. Line 82 – abbreviation mRIC is used before it is defined. It is defined on line 90.
b. Line 198 – consider changing "The CuI of TRM at day 100 and 1-year CuI was …" to "The CuI of TRM at day 100 and 1-year was …"

2. Figure 1 – interesting figure, a lot of information in a small area, but not sure how much it adds honestly.

Version 1:

Reviewer comments:

Reviewer #1

(Remarks to the Author)
The authors adequately addressed many of the issues raised by this reviewer

I still do not see how cytokine levels clearly tie into patient outcomes and were those levels unique to this disease or this regimen.

Also why is the TRM so high patients over 60 with this regimen? This is not the experience of single centers nor in registry data in EIC protocols (understanding that the HCT CI might have been high in this group and that PTCL pr present their own unique challenges. Is this thought to be a consequence of the regimen in older patients and how would authors address this in the future?

Reviewer #2

(Remarks to the Author)
The authors have adequately addressed the majority of the concerns in their response document. However, the absence of a document with tracked changes makes it difficult to identify exactly where modifications to the original submitted draft have been incorporated. We would encourage the authors in the future to ensure that revisions include a document with tracked changes, or else to identify in their response to reviewers exactly where revisions have been made in the original manuscript draft.

Following a re-read of the modified manuscript, we only have a few additional minor revisions to recommend.

1. Supplemental Table 2 is important, and we ask that it be included among the main tables of the manuscript. Alternatively, consider adding to Figure 3 a few additional PFS and OS plots visually representing the most salient data in supplemental Table 2. This could help the reader judge for themselves in which patients they might want to choose or avoid for the regimen described in this manuscript, due to the risk of early TRM. While we understand that the total number of treated patients is small and studies are ongoing, we believe it is important for the reader to see the current data.

2. While we agree that the data suggests a possibly important GvT effect, and while we appreciate that the authors softened some of their statements on GvT as part of revisions, we still believe that the data would suggest that any statements about GvT must be counterbalanced by acknowledgments of the risk of early TRM. As practicing clinicians, we have asked ourselves whether we would implement the regimen described in this manuscript in our own patients with refractory PTCL. Based on the data, for instance in older patients over age 60, the high risk of early TRM would make us think carefully about whether the risk justifies whatever GvT effect may be present. We therefore ask that the authors include in the concluding paragraph of the discussion one or more statements about who may most benefit from their therapeutic platform, and in whom it might be avoided due to the high risk of early TRM.

Reviewer #3

(Remarks to the Author)
I co-reviewed this manuscript with one of the reviewers who provided the listed reports. This is part of the Nature Communications initiative to facilitate training in peer review and to provide appropriate recognition for Early Career Researchers who co-review manuscripts.

Reviewer #4

(Remarks to the Author)
Review comments all addressed.

Version 2:

Reviewer comments:

Reviewer #1

(Remarks to the Author)
The authors have responded as well as I suspect they can to most recent comments

Reviewer #2

(Remarks to the Author)
The authors addressed our concerns. We have no further comments.

Reviewer #3

(Remarks to the Author)

I co-reviewed this manuscript with one of the reviewers who provided the listed reports. This is part of the Nature Communications initiative to facilitate training in peer review and to provide appropriate recognition for Early Career Researchers who co-review manuscripts.

Open Access This Peer Review File is licensed under a Creative Commons Attribution 4.0 International License, which permits use, sharing, adaptation, distribution and reproduction in any medium or format, as long as you give appropriate credit to the original author(s) and the source, provide a link to the Creative Commons license, and indicate if changes were made.
In cases where reviewers are anonymous, credit should be given to 'Anonymous Referee' and the source.
The images or other third party material in this Peer Review File are included in the article's Creative Commons license, unless indicated otherwise in a credit line to the material. If material is not included in the article's Creative Commons license and your intended use is not permitted by statutory regulation or exceeds the permitted use, you will need to obtain permission directly from the copyright holder.
To view a copy of this license, visit https://creativecommons.org/licenses/by/4.0/

REVIEWER COMMENTS

We greatly appreciate the reviewers' comments and have made substantial changes to the manuscript, figures, and tables accordingly. We feel this improves the manuscript and are thankful for the opportunity to submit this revision.

Reviewer #1 (Remarks to the Author):

The authors present results from a prospective trial of allo-transplant for patients with a wide variety of T and NK cell lymphomas. They utilize a platform that incorporates distal horse ATG and PTCy as part of the treatment schema. Results demonstrate PFS of over 50% in what appears to be a relatively high risk group of patients which is at least as good as that presented in prior reports. The regimen was rather toxic however with 15 of 31 patients requiring intubation and 5 requiring renal replacement therapy. It does appear that patients with what was termed refractory disease could achieve prolonged remissions with this approach.

1) Toxicity and non relapse mortality were relatively high. Do the authors attribute this to the regimen or clinical status of the recipients?
We attribute the relatively high TRM and toxicity to the pre-HCT clinical status of many recipients. As a starting place, the median HCT-CI score was 4, which does prognosticate a high TRM. While it is still a high number, 12, not 15, patients required intubation and we have changed the word "twelve" to "12" in the manuscript to make this stand out better from the "15" earlier in the same sentence (15 went to the ICU). The *very early* TRM is the most notable toxicity and, based on the reviewer's comments, we dissected the HCT-CI scores to look more at the cardiopulmonary risk (which likely contributes to risk of ICU stays and intubation post-HCT). We have included these pre-HCT risk comparisons of younger and older adults in a new supplemental figure, Suppl Figure 2. Additionally, we have added more exploratory subgroup survival analyses to Suppl Table 2 - nodal vs non-nodal disease, by HCT-CI score, and active disease vs PR/CR.

Active disease at time of HCT was also likely a factor, increasing overall inflammation and complications/toxicity. We transplanted a high number of patients with active disease causing systemic dysfunction (extranodal disease, leukocytosis/eosinophilia, elevated inflammatory markers, etc). It is possible that other centers would have reported some of the TRM deaths on this study as relapse-related deaths given the presence of active disease. We intentionally decided to consider these deaths TRM to better reflect the circumstances of the death to the readers and keep the data clean. We have an essentially parallel alloHCT study using nearly this same platform in patients with non-malignant T cell dysregulation due to "hard to engraft" inborn errors of immunity as their indication for HCT. Across these 25 patients with a *younger* median age of 24 years, the 1-year OS is 84% with *only 2 TRMs*. The data from this parallel study does inform our understanding of the factors related to TRM in the PTCL alloHCT study. We do plan to combine some of the data from the two parallel studies (65 patients total) to have larger numbers to delve further into the cytokine profiles, ATG levels, and immune reconstitution. The data from the inborn error of immunity study will mature enough for publication in 8-12 months.

We did also have a cluster of early deaths during the COVID pandemic, at a time when there were more transplant delays, less experienced/travel nurses, more issues with peri-engraftment volume management, delays getting diagnostic imaging and procedures, etc. As conducting this study (particularly the mRIC arm) largely during the COVID pandemic was not insignificant, we did include the year of transplant in many of our data tables.

2) What was the purpose of the ATG in this trial? Was it to promote engraftment, prevent, GVH, or deplete tumor cells pre-transplant. How do the authors assess the impact, positive or negative of the ATG as levels were low at Day 0.
We have added our hypothesis/rationale for the ATG to the methods, including also a statement about the GVH approach not considered to include the ATG. We have refined the discussion about the distally-time horse ATG.

3) The authors report that compared to younger patients, patients >60 years had higher day +7 levels of IL-8 (p=0.03) and CXCL9 (p=0.05), with a tendency toward elevation in day +7 IFN-γ (p=0.07). What impact do the authors think that has on the observed clinical findings?
We have added a figure (Supplemental Figure 4) looking at serial IL-8 levels, comparing younger vs older, those with acute GVHD vs not, early TRM vs not, and requiring immunosuppression beyond ppx vs not, finding some interesting differences in IL-8 early post-HCT. We have added literature from Dr. Stella Davies's (Dole et al, 2018 BBMT abstract) group on IL-8 and NET release and expanded our interpretation of our findings in the discussion. Once we have 65 similarly treated patients enrolled/treated across our two parallel studies, we plan to use these larger sample sizes to further analyze these cytokine data with regard to clinical outcomes.

4) In the Discussion, the authors state a recommendation that "investigators from Johns Hopkins advocate PBSC is the preferred graft source in PTCL and we echo that recommendation." Do the authors have evidence from their own data supporting this?
We have removed the phrase "we echo that recommendation" and instead added a sentence to the discussion to explain the risk/benefit analysis that led to PBSC being chosen for this study. As a side note to the reviewers, in one of our more recent opened/accruing studies (in a different patient population), we attempted to use this distal ATG RIC regimen with marrow grafts and it did not work – we had the first 2 patients experience secondary graft failure as a result of split chimerism (100% myeloid, 0% T). That study went through clinical hold and now is re-opened with PBSC grafts.

5) As the most striking results from this report are those in the 19 patients with refractory disease, please provide more data about these patients.
We have added a table that outlines each patient's key characteristics as well as their clinical outcomes (Supplemental Table 3), as well as added OS and PFS by disease status to Supplemental Table 2.

6) The manuscript, particularly the discussion could be shortened significantly.
We attempted to shorten the manuscript but given the insightful/thoughtful feedback from other reviewers asking for additional information, we could not significantly shorten the manuscript.

We did shorten the first paragraph of the discussion. Reviewer 4 felt the manuscript was well-written and concise, so we considered that input as well.

Reviewer #2 (Remarks to the Author):

Comments to the authors:
Rechache et al describe a prospective clinical trial in patients with PTCL, including patients with progressive disease at the time of hematopoietic cell transplant (HCT), targeting a patient population in whom there is a paucity of prospective HCT-based studies. The approach taken is aimed to address the challenge of progressive disease prior to HCT, and while it does show some promise it is also associated with significant incidence of early transplant-related mortality (TRM). This makes it difficult to immediately translate the findings into practice, and would require further study to best ascertain which patients would be most suitable for this sort of transplant. In our opinion, the overall effort is commendable and the methodology is sound. The manuscript can be acceptable for publication after major revisions, and would be suitable for a journal for HCT providers treating PTCL.

Specific comments are below:
Major Comments
1. It is commendable for the authors to provide an approach for patients who otherwise would not have access to curative therapy. Unfortunately there was a high incidence of early TRM, particularly for a regimen that is considered to be one of reduced intensity (RIC). The authors should put into context how this incidence of early TRM compares to most RIC regimens based on retrospective registry data.
Reviewer #1 had the same comments – please see answer to Reviewer #1, Q#1, above. We did consider including more comparative literature in the paper, but found ourselves taking significant space to explain to the reader why each study was or was not comparable (only younger pts, only remission pts, use of MAC, inclusion of T-ALL, no use of haplo donors, etc) and decided that this level of caveated partial comparisons was not helpful to the reader. Also, as we mentioned to reviewer #1, it is likely that TRM in our study is higher because other studies would have categorized these patients as having relapse-related events as a competing risk for TRM. For these reasons, we have left the discussion with a select group of studies that quote TRM from 12% to 21% to 27%. We are also acutely aware that the clinical outcomes for PTCL pts undergoing transplant are understudied and look forward to the results of the disease-specific HCT outcomes for a range of PTCL histologies that CIBMTR is currently working on (one of which Dr. Rechache is PI).

The high early TRM makes it challenging to integrate the approach described into standard practice. The authors should present additional PFS and OS curves, dichotomizing by disease type (for example, combination of AITL, PTCL-NOS, Alk-neg ALCL vs the rest; PTCL with progressive disease at the time of HCT vs the rest), age (< 60 vs >= 60) and HCT-CI score (0-3 vs > 3) in order to provide more insight as to what baseline characteristics may have been associated with outcomes. The authors note there was a difference in TRM between patients age < 60 vs >= 60, but it is not clear why this would be the case for a RIC regimen – did the older patients have more aggressive disease, more prior therapies, more baseline comorbidities? Were there any specific subcomponents of the HCT-CI (for example baseline cardiovascular

comorbidities, pulmonary comorbidities, etc) that were associated with TRM? Is there a patient subgroup that would have the greatest benefit relative to risk from the approach described in this manuscript?
We have added outcomes by disease status, nodal vs extranodal disease, and HCT-CI score to Supplemental Table 2, our exploratory subgroup analyses of OS and PFS. Please also see response to Reviewer #1, Q#1 regarding differences between the older and younger patients. We have also added that younger patients would likely have the greatest benefit to the discussion.

2. We note that an important limitation of the data is that due to the high incidence of early TRM, it is difficult to make any statements about the graft-vs-tumor effect. Since an evaluation for disease relapse could not be done at the time of clinical decompensation, and since the latter was often early, could it have been the case that many patients with active disease at the time of HCT would have eventually relapsed had they not died for another reason? The authors should soften their statements about the graft-vs-tumor effect throughout the manuscript given it could not be evaluated in a sizeable portion of the treated patients.
We agree this is an important limitation to not know if more patients with active disease would have relapsed or been cured. However, we are struck by the 10 survivors who were transplanted with refractory, active disease that would have led to their death in weeks to months without transplant. While we fully appreciate the limitations, the GVT seen in this study is the most compelling finding and could lead to practice change, with more movement of patients with active disease to HCT. Nonetheless, we have softened some of the GVT language in the manuscript. We do think it is important to keep the caution about post-HCT immunosuppression in the discussion of how to harness GVT.

3. As we understand it, the hypothesis with the use of hATG was to provide some measure of disease burden reduction alongside the other conditioning regimens, and with aim to have cytotoxic levels clear out before day 0. But it is not clear what is a 'therapeutic level' and what is a 'cytotoxic level' of hATG based on the presented data. The authors should indicate these levels in Figure 5 and provide additional references for these levels. Furthermore, many HCT protocols use ATG, and many give it more proximally to day 0. Please explain how the data on eATG levels with the more distal administration compares to other protocols where the ATG is given more proximally.
Reviewer #1 had this same question (see response to Reviewer #1, question #2). Unlike other groups who use ATG for the dual purpose of conditioning and GVHD ppx, we were aiming for levels as low as possible at day 0.

4. A recent publication (Admiraal R et al, Blood Advances 2025) indicated that model-based dosing of ATG can lead to improved survival and reduced morbidity, with the model based in part on the ALC. The authors show the eATG levels relative to ALC pre-HCT in Figure 5B, but there does not appear to be any difference based on the baseline ALC – please discuss this in the context of the recent Blood Advances paper.
We have added the Admiraal citation to the discussion and added more to the discussion of how pre-HCT factors might inform distal ATG in future studies.

5. It is notable as well that the cumulative incidence of severe chronic GVHD of 23% is high for PTCY-based regimens with RIC conditioning. One possibility is that the early immune

suppression discontinuation, while aiming to promote a graft-vs-tumor effect, came at a significant cost. The authors should describe the organ manifestations in the patients with chronic GVHD. Among surviving patients beyond 1 year, what was the incidence of severe chronic GVHD? In addition, according to the text there were 11 patients in whom sirolimus was stopped even earlier than the planned day +60. The authors should provide an explanation as to why this was, and conduct a subgroup analysis comparing outcomes of these patients relative to others with respect to PFS, OS, TRM, and incidence and severity of chronic GVHD.
We have added a table that shows outcomes for each patient (per the request of reviewer #1, Q#5) and have included organ involvement for those with cGVHD in that table (Supplemental Table 3).

6. The 60-day cumulative incidence of graft failure of 10% is important to discuss. Please discuss this in the context of graft failure rates in HLA mismatched transplants in general.
The GF is slightly higher than would be expected for RIC but we believe these GFs were situational and not platform-related (hence the info added about DSAs, cryopreservation issues, etc). We have also added graft characteristics column to Supplemental Table 3.

7. In supplemental Table 2, there appears to be a difference (though not clear if statistically significant based on small numbers) between outcomes of HLA matched and HLA mismatched transplants. What is the hypothesis for this?
The analysis of survival outcomes by HLA match was indeed not significant. However many of those who received haplo grafts were also older and we know that the older patients had more TRM. Thus, we do think that these variables are colinear and the true risk factor is age. However, there was more CRS in HLA-mismatched transplants, so this may have contributed to some degree. Even when the study is complete and the numbers are bigger, it is unlikely that we will be able to conduct multivariate analyses. We have added more analyses of IL-8 levels, by pre-HCT risk scores, etc (Supplemental Figure 2, Supplemental Figure 4).

8. It was hypothesized that filgastrim in the context of the mRIC regimen may augment the clearance of hATG-bound cells, but this did not appear to be the case as per the data in Figure 5A. So then why in the comparison of PFS between the two arms does there appear to be a visual difference between RIC and mRIC(Figure 2A), with a seemingly rapid drop in the PFS curve in the mRIC arm? The authors should comment on this.
Figure 5A (now Supplemental Figure 6, due to reorganization) shows total levels, not clearance. We would not expect total levels to be different by arm. The rapid drop in PFS in mRIC is related to the early TRM previously explained (sepsis, volume overload, etc) and differences in age, HCT-CI score, etc between the RIC arm and mRIC arm. Thus, we do not think the addition of G-CSF in the mRIC arm is the factor that leads to the survival differences, but rather other variables that differ between arms.

9. To add to the point above, what is the hypothesis about the differences in reconstitution of T and NK cell populations between RIC and mRIC given that up-front filgastrim did not augment the clearance of hATG-bound cells?
By day 0 we do not see much ATG around in either arm so do not think it affects IR. There was no difference in the level of IR by study arm, CMV-serostatus, or HLA-match. Given that there are no differences and the fact that Figure 4 (now Supplemental Figure 5) is busy, we have

consolidated the box and whiskers at each timepoint to show all patients, leaving the comment of no difference in IR between subgroups in the results text.

Minor Comments

1. Please put the number of patients evaluated at each time point in each figure panel as it helps to consider how many were at risk for a particular event. This was done in some plots and not others. Please do this for all survival curves. ->Updated.

2. The fonts have to be consistent across all plots. For example, in Figure 3, the font sizes appear different in each subplot.

->Adjusted font sizes.

We can barely read some of the numbers. Figure 3D should go in the supplemental as it is only showing data for 1 patient, and this can help with the font sizes in the other plots. Please re-make the figures and use a consistent font size across all plots. This may mean putting some more of them in the supplemental. ->Adjusted font sizes.

3. Figure 4 could go into the supplemental, and again, please remake the plots so that the fonts are not so tiny. We cannot tell if there was any statistical analysis done in these figures to compare the RIC and mRIC arm. No significant differences, now Supplemental Figure 5 with revisions (per #9, above)

4. We suggest for the swimmer's plot (Figure 6) to be moved up earlier, possibly as Figure 2.We agree this improves the order in which data are presented and have moved to Figure 2.

5. Supp Figure 2: this figure needs to be revised. The font is way too small. Put "n=" as an indication of the number of patients evaluated under each time point.

Adjusted figure. Now Supplemental Figure 3.

6. Supp Figure 3: the font is way too small. We think there were some statistical comparisons done, but we can't tell what is significant or not.

Adjusted figure. Now Supplemental Figures 7-8.

Reviewer #4 (Remarks to the Author):

Summary:

This manuscript decribes a single arm, single institution, prospective experience using allo-HCT to treat T and NK cell lymphomas. The majority of these patients had high risk disease, many had persistent disease even at high levels. This was an overall high risk cohort in respect to comorbid conditions and need for mismatched donors. The first group of patient had a RIC allo as described in the paper, and the second cohort with a modified RIC platform with GCSF to

modulate effect of ATG.

Correlative studies including immune reconstitution by flow cytometry and serum cytokines were done.

Results were promising, underscoring the graft versus tumor effect in this disease, and the fact that good outcomes can be attained even in patients with active disease. The TRM was concerningly high in the older patients, which likely renders this platform best used in younger patients.

Correlatives were well done and communicated. However, I would say there were relatively few clear signals from the analysis, which is common for transplant studies. This reflects how challenging it is to do and analyze these data and also the differences in the biology of patients before and peri-transplant. But it is important to continue to do these studies as we get more insight into post transplant biology in the context of relapse, GVHD, and long remissions.

This manuscript has several strengths. It is remarkably well written and concise. Relevant literature is reviewed. This platform is very promising in a disease that can be difficult to treat and effect young people. The patient population was high risk, more underscoring the good outcomes.

I believe that this paper is appropriate for publication with few edits.

Major:
1. Figure 2 – I think this composite figure is misleading. Looking at Penal !, I expected the rest of the figures to be red RIC and blue mRIC. I think it is most important to differentate panel A from the others, since in reading the manuscript you expect comparisons to be RIC vs mRIC, which is not the case for any other panels. Each other panel makes a different comparison. I suggest changing he colors for panels B C and D. I also suggest having the legend in the exact same place in all four panels and making it bigger.

I understand why the composite figure is being used, but I would be careful to make it abundantly clear that all these comparisons are not RIC vs mRIC.
Adjusted figure with coloring changed to make this more clear. Now Figure 3.

2. Figure 3 – the colors don't work. In most places in panels B and C there is no color applied to the bars, since the bars and nonexistant. Adjusted figure. Now Figure 4 with color vs symbol use fixed.

3. For the CXCL9 and IL8 elevations in older patients, any correlation to the ones who had poor outcomes?
We have greatly expanded our analyses of IL-8, which some interesting early findings that we will look to understand further with larger numbers (new Supplemental figure 4)

4. Please add a sentence on why ATL patients were excluded on the mRIC arm.

We have added a sentence to the manuscript. In brief for the reviewer, there was an ATL program at the NIH with translational research findings that emerged at the time that the mRIC study expansion was being crafted. Given the aggressive nature of ATL disease *and* the unique opportunity to study them separately, we decided to not include them in the mRIC arm and design an ATL-specific arm for the study at the same time we were adding the mRIC arm.

5. Was tocilizumab used appropriately? For example, in patients with grade 2 CRS or perhaps persistent fevers? We often find it is used per MD discretion and at times in patients with grade 1 CRS but not in patients with grade 2 CRS.
Yes, tocilizumab was used per CRS standard guidelines when clinically indicated, except in one patient who received it prophylactically per recommendation of cardiology. As mentioned in the manuscript, there were other times tocilizumab was used for non-CRS reasons, based on literature in IPS, for example.

a. Why were toci patients excluded from serum IL-6 measurements? Does not need to be in the manuscript necessarily but curious what your cytokine panels revealed. Were IL-6 levels higher?
Because tocilizumab is a competitive inhibitor of IL6 receptor, IL-6 levels can increase dramatically after toci administration. We discussed with others at NCI how to adjust for this intervention and followed the practice of one of our CART colleagues, Dr. Nirali Shah, who advised excluding IL-6 levels collected in the 7 days post-toci, aligning our method with her published method of IL-6 level data analysis

6. In the Supplement, sample size for RIC arm was 21 (enrolled) and sample sie for mRIC arm was 20. Why did you publish early? Is the trial still enrolling? Please clarify in the manuscript.
Prior to this manuscript being sent for review, the editors did also clarify the reason for interim analysis and this was provided to their satisfaction. In brief, there were 3 early deaths in rapid succession on the mRIC arm and, even though stopping rules were not activated at that time, the PI opted to pause and analyze the data. For a variety of reasons, the pause and analysis then became more extensive, even looking at some of our exploratory endpoints, leading to a year long pause in accrual. By the time the study reopened, this group of 31 had completed follow-up for the primary endpoint and thus are being published now, along with the extensive data analysis that was done that allows the RIC arm to be compared to the mRIC arm.

Minor:
1. Grammatical issues:
a. Line 82 – abbreviation mRIC is used before it is defined. It is defined on line 90.
b. Line 198 – consider changing "The Cul of TRM at day 100 and 1-year Cul was …" to "The Cul of TRM at day 100 and 1-year was …"
Done

2. Figure 1 – interesting figure, a lot of information in a small area, but not sure how much it adds honestly. Given that other reviewers asked for more information about the patients, particularly those with refractory disease, we have opted to keep this figure in the manuscript but modified it for greater readability and understanding.

We thank the reviewers for their thoughtful feedback. Please see our responses in red below. We hope we have adequately addressed the concerns.

REVIEWER COMMENTS

Reviewer #1 (Remarks to the Author):

The authors adequately addressed many of the issues raised by this reviewer

I still do not see how cytokine levels clearly tie into patient outcomes and were those levels unique to this disease or this regimen.

Evaluation of cytokine levels was one of the exploratory endpoints of this prospective study, intended to be hypothesis-generating for future research. Thus, the clinical significance of the cytokine levels is an evolving but potentially clinically-relevant story, especially as we try to better understand the dynamics of cytokine release peri-engraftment and over time. No definitive conclusions can be made regarding the association of cytokine levels to patient outcomes at this time, but these findings have fueled areas of future inquiry for ourselves, as well as others reading the manuscript. We intend to further study the relationship between an early hyperinflammatory state and early mortality, potentially also looking for interventional maneuvers based on these preliminary findings.

We have added the wording "exploratory" to the discussion of cytokines to make clear the intention of these investigations at this time

Also why is the TRM so high patients over 60 with this regimen? This is not the experience of single centers nor in registry data in EIC protocols (understanding that the HCT CI might have been high in this group and that PTCL pr present their own unique challenges. Is this thought to be a consequence of the regimen in older patients and how would authors address this in the future?

We think the high TRM in patients over 60 is related to several factors. Among the 9 patients >60, median HCT-CI score was higher in those who died vs in survivors (7 vs 5), prior lines of therapy were greater (5 vs 3), prior autoHCT was more frequent (60% vs 0%), leading to an overall more heavily pretreated group. Additionally, AITL with progressive disease comprised the majority of these patients with early mortality, and we have noted a preponderance of serositis associated with GVT in AITL patients in our cohort. All patients >60 who died received haplo grafts and all patients >60 who survived received matched grafts. Thus we hypothesize that 1) the underlying disease with increased preponderance for inflammation and actively progressing, 2) augmented by increased cytokine release (as evidenced by day +3 cytokine levels) in the setting of

a mismatched graft with large amounts of antigen, and 3) compounded by reduced tolerance of peri-engraftment fluid shifts due to underlying cardiopulmonary comorbidities, together contributed to the outcomes presented. We definitively do not think the principal issue is related to mismatched grafts, but rather the combination of all of these factors, and we have shown in Supplemental Table 2 (which will be added to the main manuscript as recommended by Reviewer #2) that recipients of haploidentical grafts aged <60 (n=14) had a 3yr OS of 71%.

Finally, we would like to note that it is difficult to compare the TRM in this subcohort of patients >60yo with the overall TRM in prior studies, not just due to the risk factor of older age but also given the proportion of our older patients with active disease skews much more toward progressive disease (78%, remainder in PR2) than in any prior study. These patients for the most part had few remaining treatment options offered to them, and were overwhelmingly likely to die of their disease in a matter of months if not transplanted.

In terms of how to address the high TRM in our subcohort of older patients, we have several thoughts:

1. risk factors in some of these patients may have been partially mitigated in the setting of earlier referral and/or upfront allo prior to auto leading to less pretreatment to exacerbate comorbidities. This is alluded to in the discussion (newly added text in italics): "Remission should not be required to proceed to HCT for PTCL, particularly if remission seems improbable and the patient is young, as further chemotherapy will likely introduce added toxicity *thus increasing TRM risk related to comorbidities*, while delaying allogeneic HCT."

2. older patients with cardiopulmonary comorbidities may tolerate the peri-engraftment period more successfully if strategies to mitigate cytokine release are used more proactively, either with more aggressive treatment or by selecting a matched donor, if available (typically unrelated as siblings of these patients are commonly elderly as well). This is alluded to in the discussion (newly added text in italics): "The cytokine elevations in IL-8 and the IFN-γ pathway in older patients on day +7, after PTCy abrogated clinical CRS, are of note as a direction for early intervention to decrease peri-engraftment cardiopulmonary toxicities, *particularly in recipients of mismatched grafts*."

Reviewer #2 (Remarks to the Author):

The authors have adequately addressed the majority of the concerns in their response document. However, the absence of a document with tracked changes makes it difficult

to identify exactly where modifications to the original submitted draft have been incorporated. We would encourage the authors in the future to ensure that revisions include a document with tracked changes, or else to identify in their response to reviewers exactly where revisions have been made in the original manuscript draft.

We have provided a version with tracked changes for the initial revision and the current revision and regret not including this in the prior submission.

Following a re-read of the modified manuscript, we only have a few additional minor revisions to recommend.

1. Supplemental Table 2 is important, and we ask that it be included among the main tables of the manuscript. Alternatively, consider adding to Figure 3 a few additional PFS and OS plots visually representing the most salient data in supplemental Table 2. This could help the reader judge for themselves in which patients they might want to choose or avoid for the regimen described in this manuscript, due to the risk of early TRM. While we understand that the total number of treated patients is small and studies are ongoing, we believe it is important for the reader to see the current data.

We agree that Supplemental Table 2 is informative, and have added it to the main manuscript. We have also added the 1y OS details for each group for greater clarity.

2. While we agree that the data suggests a possibly important GvT effect, and while we appreciate that the authors softened some of their statements on GvT as part of revisions, we still believe that the data would suggest that any statements about GvT must be counterbalanced by acknowledgments of the risk of early TRM. As practicing clinicians, we have asked ourselves whether we would implement the regimen described in this manuscript in our own patients with refractory PTCL. Based on the data, for instance in older patients over age 60, the high risk of early TRM would make us think carefully about whether the risk justifies whatever GvT effect may be present. We therefore ask that the authors include in the concluding paragraph of the discussion one or more statements about who may most benefit from their therapeutic platform, and in whom it might be avoided due to the high risk of early TRM.

Reviewer #1 had similar concerns regarding the TRM, and we have provided some additional insight as to contributing factors and mitigation above, along with some edits within the text. In our current practice, when counseling prospective patients over 60 yo regarding enrollment, we discuss the concern for increased mortality particularly when co-factors of cardiopulmonary comorbidities, active disease, and mismatched grafts are present. Of note, all 4 patients >60yo who received matched unrelated grafts are alive and progression-free, and we believe the high TRM in this subgroup was mediated by a combination of factors including underlying disease, comorbidities, and increased

cytokine release in the setting of mismatched grafts. We have added these clarifications in the results section (newly added text in italics): "Notably, the CuI of TRM at 1-year was 11% (95% CI 2-29%) for patients ≤60 years but significantly higher at 56% (95% CI 17-82%) for patients >60 years, p=0.01 (Figure 3d), *the latter exclusively in recipients of mismatched grafts, who also had greater comorbidities and were more heavily pretreated*." Updated in the second to last concluding paragraph of the discussion (newly added text in italics): "While the PFS, relapse, OS, and severe acute GVHD rates are overall promising, the high rates of early TRM in older patients warrant further consideration to improve the platform. The cytokine elevations in IL-8 and the IFN-γ pathway in older patients on day +7, after PTCy abrogated clinical CRS, are of note as a direction for early intervention to decrease peri-engraftment cardiopulmonary toxicities, *particularly in recipients of mismatched grafts*."

In the final paragraph, we mention young age and fewer comorbidities as preferred factors in proceeding to HCT for patients with refractory disease (newly added text in italics): "Remission should not be required to proceed to HCT for PTCL, particularly if remission seems improbable and the patient is young, as further chemotherapy will likely introduce added toxicity *thus increasing TRM risk related to comorbidities*, while delaying allogeneic HCT." We also allude to alternative ATL-specific approaches under study, as both ATL patients transplanted with this regimen died: "We continue to prospectively study allogeneic HCT for PTCL, including ongoing study of ATL-specific approaches with promising early results."

Apart from the above updates and highlights related to age, comorbidities, HLA-match, and disease specifics (ATL vs non-ATL), including the addition of the subgroup analyses to the main manuscript, we believe adding further specifics to the text, regarding which patients the platform is best suited to, is premature based on the small size of the subgroups.

Reviewer #3 (Remarks to the Author):
I co-reviewed this manuscript with one of the reviewers who provided the listed reports. This is part of the Nature Communications initiative to facilitate training in peer review and to provide appropriate recognition for Early Career Researchers who co-review manuscripts.
Thank you for your review.

Reviewer #4 (Remarks to the Author):
Review comments all addressed.

Thank you for your review.
